# Decidual-Placental Immune Landscape During Syngeneic Murine Pregnancy

**DOI:** 10.3389/fimmu.2018.02087

**Published:** 2018-09-19

**Authors:** Yan Li, Gladys E. Lopez, Jessica Vazquez, Yan Sun, Melina Chavarria, Payton N. Lindner, Samantha Fredrickson, Nathan Karst, Aleksandar K. Stanic

**Affiliations:** ^1^Division of Reproductive Sciences, Department of Obstetrics and Gynecology, University of Wisconsin-Madison, Madison, WI, United States; ^2^Division of Reproductive Endocrinology and Infertility, Department of Obstetrics and Gynecology, University of Wisconsin-Madison, Madison, WI, United States; ^3^Reproductive Medicine Center, Fujian Provincial Maternity and Children's Hospital, Affiliated Hospital of Fujian Medical University, Fuzhou, China

**Keywords:** dendritic cells, T cells, t-SNE, decidua, placenta, C57BL/6 mice

## Abstract

Adaptive immune system, principally governed by the T cells—dendritic cells (DCs) nexus, is an essential mediator of gestational fetal tolerance and protection against infection. However, the exact composition and dynamics of DCs and T cell subsets in gestational tissues are not well understood. These are controlled in human physiology by a complex interplay of alloantigen distribution and presentation, cellular/humoral active and passive tolerance, hormones/chemokines/angiogenic factors and their gradients, systemic and local microbial communities. Reductive discrimination of these factors in physiology and pathology of model systems and humans requires simplification of the model and increased resolution of interrogative technologies. As a baseline, we have studied the gestational tissue dynamics in the syngeneic C57BL/6 mice, as the simplest immunological environment, and focused on validating the approach to increased data density and computational analysis pipeline afforded by highly polychromatic flow cytometry and machine learning interpretation. We mapped DC and T cell subsets, and comprehensively examined their maternal (decidual)—fetal (placental) interface dynamics. Both frequency and composition of decidual DCs changed across gestation, with a dramatic increase in myeloid DCs in early pregnancy, and exclusion of plasmacytoid DCs. CD4+ T cells, in contrast, were lower at all gestational ages and an unusual CD4^−^CD8^−^TCRαβ^+^group was prominent at mid-pregnancy. Dimensionality reduction with machine learning-aided clustering revealed that CD4^−^CD8^−^ T cells were phenotypically different from CD4+ and CD8+ T cells. Additionally, divergence between maternal decidual and fetal placental compartment was prominent, with absence of DCs from the placenta, but not decidua or embryo. These results provide a novel framework and a syngeneic baseline on which the specific role of alloantigen/tolerance, polymicrobial environment, and models of pregnancy pathology can be precisely modeled and analyzed.

## Introduction

Pregnancy is a remarkable challenge that requires coordination of multiple systems, including adaptive and innate immune cells, systemically and at the maternal-fetal interface. Immune cells in pregnancy are responsible for fetal protection from pathogens, establishing and maintaining tolerance for the semi-allogeneic fetus, and directing the placental remodeling of uterine vasculature (1). Critical decisions regarding the type and intensity of adaptive immune response are primarily driven by the decisions reached by T cells interacting with dendritic cells (2), the binary cellular focus of this study. Rich diversity of immune cellular phenotypes and a multitude of influences (chemokines, developmental cytokines, allostimulation in non-syngeneic pregnancies, and microbiome of the reproductive tract) makes this a particularly challenging, yet pivotal, system to study (1, 3, 4). Advances in immunology and a deeper understanding of tissue resident immune cell dynamics in other contexts warrant a re-examination of gestational adaptive immune dynamics (5–7). To manage complexity, and to establish a higher resolution approach to adaptive immune dynamics, we applied high-dimensional flow cytometry and a machine learning pipeline to a simplified model of pregnancy—syngeneic C57BL/6 cross. Herein, allostimulation, complex microbial challenge and polygenic diversity is restricted, thus, allowing us to test the application of high-dimensional immunology in gestational tissues, to set the stage for next generation of complex studies that would reflect human and mouse pregnancy physiology.

DCs (CD11c^+^) participate in establishment of maternal immunologic tolerance (8) and pregnancy establishment more broadly (1). During embryo implantation DCs may promote angiogenesis, a process necessary for adequate spiral artery remodeling in mice (9, 10). Indeed, depletion of uterine DCs in a CD11c-DTR transgenic model causes failure of decidualization, impaired implantation, and embryonic resorption (8, 11). DCs (identified as CD11c^hi^MHCII^+^F4/80^−^) are trapped within the pregnant uterus in mice, inhibiting antigen transfer to the local lymph node, a process necessary for priming of circulating naïve T cells (12), presumably in order to prevent alloantigen anti-fetal response. Despite this trafficking restriction, DCs can prime CD8+ T cells, as CD11c+ depletion abrogated the CD8+ T cells response to infection with *Listeria monocytogenes* and *Plasmodium yoelii* in a mouse model (13). Despite these intriguing studies, surface receptor heterogeneity of DCs subsets in peripheral tissues and side-effects of diphtheria toxin receptor (DTR)-based depletion methodology impose significant limitations on interpretation of this data. For one, the CD11c-DTR mouse model is not wholly DCs specific, as certain macrophages, plasmablast, activated T cells, and NK cells, can express DTR and be at least partially depleted, with mouse morbidity and death after repeated DT injections (14, 15).

Multiple T cells subsets perform a variety of functions (16, 17) in pregnancy. Regulatory T cells (Tregs) maintain tolerance toward the semi-allogeneic fetus in mice (18, 19). Tregs deficiency impedes implantation in mice either due to implantation failure or embryo resorption (16, 20). Tregs during pregnancy are enriched primarily at the maternal-fetal interface, but not in the circulation, highlighting the distinct phenotype and function of decidual T cells (21). Furthermore, type 1 helper (Th1) cells and cytotoxic T cells (CTLs) are actively excluded from mouse decidua, due to an epigenetic chemokine gene silencing program rather than an alloantigen-specific manner (22). However, comprehensive assessment of decidual T cell subset dynamics in the syngeneic breeding mouse model is still lacking (16). Advent of highly polychromatic flow cytometry and computational analysis methods allows for a redefinition of immune system dynamics during pregnancy given complex data in multiple models (23).

In this study, although no allo-reactivity or complex polymicrobial environment is present, the dynamics of T-DCs in this system may reveal the influence of other gestational factors (hormonal, pregnancy stage-specific chemokines, or others). Therefore, we focused on the following questions: (a) Does application of high-dimensional flow cytometry and machine learning bring a more nuanced insight into the dynamics of DC/T cells in gestational tissues? (b) What is the population dynamics of DC/T cell subsets in the uterus, decidua and placenta across syngeneic mouse gestation? This study, while not reflective of a physiological pregnancy, provides a baseline that integrated with allogenic and microbial influences will increasingly provide more detailed, complex and faithful representations of adaptive immunology of pregnancy.

## Materials and methods

### Mice

Female and male C57BL/6J (B6) mice were purchased from Jackson laboratory (Bar Harbor, ME, cat# 000664). The mice were housed in specific pathogen free facility at the Biotron in ventilated micro-isolator cages at University of Wisconsin-Madison. All caging equipment, bedding and enrichment items are sterile and mice are provided with irradiated feed and acidified water. The breeding and timed mating were set up and maintained by trained staff. The protocol was approved the Institutional Animal Care and Use Committee at University of Wisconsin-Madison. Female mice (6–13 weeks) were used for timed mating and experiments. The day when a vaginal plug was detected in a timed mating was counted as gestational day 0.5. Virgin mice (6–13 weeks) and the mice at various specified gestational day (early: 6, 7, 8, mid:12, 13, 14, late: 16, 17, 18 days) were sacrificed, and gestational day of each embryo/decidua/placenta/uterus saved for analysis.

In this study, estrous stage was not assessed in the virgin mouse group. In order to keep the experiments consistent, the mice used for timed mating and virgin controls were roomed together for weeks, with likely estrus synchrony as occurs in co-housed mice. Still, virgin mice from different individual experiment are likely to be in different stages of estrous, contributing some immune cell variability to the virigin uterus “baseline” studies.

### Tissue processing/immune cells isolation

Mouse decidua, placenta, embryos (GD6-8 only), and uteri (virgin only) were collected (Supplementary Figure 5 shows the images of dissected tissues) as guided by the book “The Guide to Investigation of Mouse Pregnancy” edited by B. A. Croy et al. For the separation of embryo and decidua, briefly, an individual implantation site was isolated by making two vertical cuts across the short axis of the uterine horn, then retracted the cut edges over the capsule permitting the visualization of the decidual capsule attachment site to the myometrium, a horizontal cut was made to isolate the decidua basalis from the placental primordium embryo(see Croy, et al. chapter 2 plate 3 J; and Supplementary Figure 5 in this study), the red steak defined the embryo, the remainder was the decidua basalis (24). All the collected tissues were subsequently minced with scissors in RMPI 1640 containing collagenase type V (Worthington Biochem, cat# LS005282)/DNAse I (Worthington Biochem, cat# LS006344). These specimens were then processed using gentleMACS^TM^ C tube (Miltenyi Biotec Inc., San Diego, CA, cat# 120-005-331), and a specially adapted tissue dissociation program (clockwise spin of 100 rpm for 1 min, then counter-clockwise spin of 100 rpm for 1 min, followed by clockwise spin of 1,000 rpm for 5 s, loop 5 times, finally counter-clockwise of 100 rpm for 1 min and then clockwise spin of 100 rpm for 1 min, all steps were within 37°C) run in gentleMACS™ Dissociator for 30 min (Miltenyi Biotec Inc. San Diego, CA, cat# 130-096-427). Spleen, thymus, and Peyer's patches (used as control tissue) were mechanically dissociated in RMPI 1640 containing 10% heated FBS in gentleMACS™ C tube, by running corresponding standard programs for different tissue types in gentleMACS™ Dissociator.

After dissociation, homogenates were filtered through 70 μm cell strainer, and red cells of splenic or thymic (as needed) specimens were lysed with ACK lysis buffer (Life Technologies, cat# A10492-01). Single cell suspension obtained was used for downstream applications.

### Flow cytometry labeling and analysis

Single cell suspensions were first labeled with LIVE/DEAD® fixable blue stain (Invitrogen, cat# L34962) and subsequently a cocktail of flourochrome-conjugated monoclonal antibodies (list in Table 1) according to the manufacturer's instructions. Briefly, antibodies were diluted in BD Horizon BrilliantTM Stain Buffer (BD Biosciences, San Jose, CA, cat#566349) and used to label cells for 30 min, washed, and fixed with 4% formaldehyde (TED PELLA, Inc., cat# 1805) for 5 min before washout using stain buffer (BD, cat#554656). Transcription factor assessment for intracellular staining was done using BD Pharmigen™ Transcription Factor Buffer Set (BD, cat# 562574). UltraComp eBeads were used for compensation (eBioscience, cat # 01-222-42).

**Table 1:** Antibodies used to label dendritic cells and T cells.

**Antibody**	**Clone**	**Flourochrome**	**Supplier**
CLEC9A	104B	BB515	BD Bioscience
CD25	PC61	BB515	BD Bioscience
I-A/I-E	M5/114.15.2	PerCP-Cy5.5	BD Bioscience
CD44	IM7	PerCP-Cy5.5	BD Bioscience
CD14	RmC5-3	PE	BD Bioscience
CD69	H1.2F3	PE	BD Bioscience
CD80	16-10A1	PE-CF594	BD Bioscience
CD62L	MEL-14	PE-CF594	BD Bioscience
CD8a	53-6.7	PE-Cy5	BD Bioscience
CD3	145-2C11	PE-Cy7	BD Bioscience
NK1.1	PE-Cy7	PK136	BD Bioscience
L-y-6G	1A8	PE-Cy7	BD Bioscience
B220	RA3-6B2	PE-Cy7	BD Bioscience
CD209	5H10	APC	BD Bioscience
CD122	TM-Beta 1	APC	BD Bioscience
L-y-6G	RB6-8C5	AF700	BD Bioscience
TCRβ	H57-597	AF700	BD Bioscience
B220	Ra3-6b2	APC-Cy7	BD Bioscience
CD11c	HL3	BV421	BD Bioscience
CD196	140706	BV421	BD Bioscience
F4/80 like receptor	6f12	BV510	BD Bioscience
CD127	SB/199	BV510	BD Bioscience
CD11b	M1/70	BV605	BD Bioscience
I-A/I-E	M5/114.15.2	BV605	BD Bioscience
BST2	927	BV650	Biolegend
CD19	1D3	BV395	BD Bioscience
CD4	GK1.5	BUV496	BD Bioscience

Data were acquired using the LSR Fortessa in a 5 laser (UV 355 nm, Violet 405 nm, Blue 488 nm, Yellow/Green 561 nm, Red 643 nm) 20-detector configuration (BD Biosciences). SPHERO TM Rainbow Calibration Particle (Spherotech Inc., cat# RFP-30-5A) were used to standardize PMT voltage settings across sequential experimental runs.

### Data analysis

Manual gating analysis was performed using FlowJo v.10.3 (Flowjo LLC, Ashland, OR). Dimensionality reduction was performed using the t-SNE algorithm, followed by DensVM clustering, both part of the open-source R package, Cytofkit (github.com/JinmiaoChenLab/cytofkit) (25). Briefly, data files were pre-gated to exclude dead cells and irrelevant lineage populations and concatenated using FlowJo. Concatenated files were then entered into the R/Cytofkit analysis pipeline via the GUI interface, and parameters of interest selected. Newly derived t-SNE and DensVM coordinates were added to original data matrices, exported, and analyzed in FlowJo. Cluster frequencies and mean fluorescence intensity (MFI) values were calculated using FlowJo and exported into Excel and JMP Pro 13 (SAS, Cary, NC, USA) for analysis. Heatmaps for MFI (z-score normalized) and cluster frequencies were constructed in JMP Pro 13.

The data from each gestational day were analyzed separately, when we created the figure and analyzed statistics, we combined the data of early pregnancy GD 6, 7, 8 together, middle pregnancy GD 12, 13, 14 together, late pregnancy GD16, 17, 18 together, respectively. Manual statistical analysis was performed using GraphPad Prism 7 (GraphPad Software Inc., La Jolla, CA, USA). One-way ANOVA followed by Tukey's multiple-testing adjusted *post-hoc* analysis was used to determine statistical significance (*p* < 0.05), all statistical analysis details are shown in Supplementary Tables 4–8. All data are represented as box plots with minimum to maximum, showing all points.

## Results

### Dramatic remodeling of DC compartment across pregnancy

**Figure 1:**
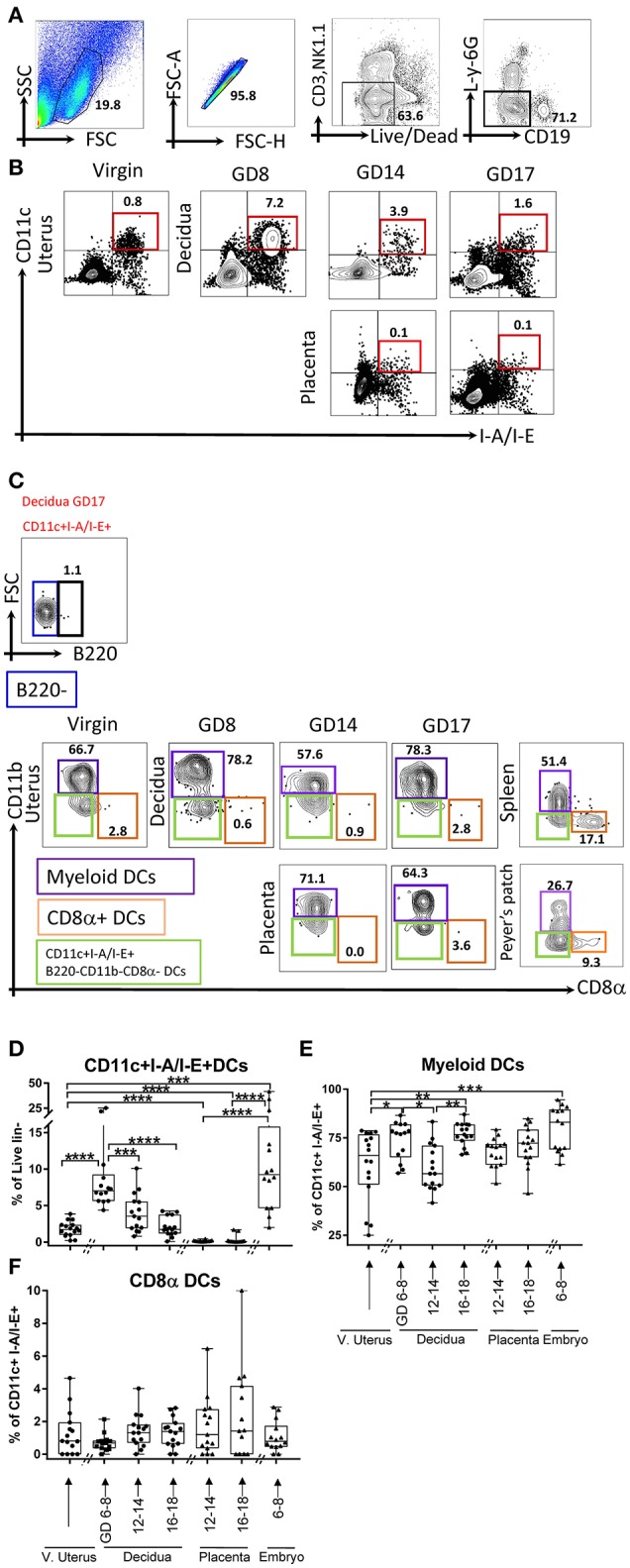
Flow cytometry gating strategy showing dynamics of dendritic cells (DCs) in the murine uterus, decidua, placenta, and embryo. **(A,B)** Dendritic cells, defined as live and lineage negative (lin1-: CD3^neg^NK1.1^neg^CD19^neg^L-Y-6G^neg^, **A**) CD11c+I-A/I-E+ **(B)**, were identified in the mouse virgin uterus, decidua (**B**, top panel), and placenta (**B**, lower panel) across mouse gestation. **(C)** The subset of DCs, Myeloid DCs (lin1-CD11c^+^I-A/I-E^+^B220^neg^CD11b^+^CD8α^neg^) and CD8α^+^ DCs (lin1-CD11c^+^I-A/I-E^+^B220^neg^CD11b^neg^CD8α^+^), were further identified in murine virgin uterus, decidua, placenta across gestation age. **(D**) Proportions of CD11c+I-A/I-E+DCs out of lin1-cells in the murine virgin uterus, decidua, placenta, and embryo across gestation age. **(E)** Proportions of myeloid DCs out of CD11c+I-A/I-E+DCs in the virgin uterus, decidua, placenta, and embryo. **(F)** Proportions of CD8α+DCs out of CD11c+I-A/I-E+DCs in murine virgin uterus, decidua, placenta, and embryo across gestation age. These gating strategy figures were not obtained from the same experiment. *n* = 14–16 (16 independent experiments), the exact number of animals used were summarized in Supplementary Table 3. The data of virgin uterus were compared with decidua group or placenta (include embryo) group, respectively. Statistical analysis was performed using ANOVA followed by *post-hoc* Tukey analysis. *p < 0.05, **p < 0.005, ***p < 0.0005, ****p < 0.0001.

To examine the composition of DC subsets across gestational age, we validated a comprehensive, polychromatic (16-marker) panel (Table 1). We monitored overall DCs (Figures 1A,B, lin1-: CD3^neg^NK1.1^neg^CD19^neg^Ly-6G^neg^, CD11c^+^I-A/I-E^+^ cells) and subsets (Figure 1C). DCs in control tissues (spleen, Peyer's patches, and thymus) were examined in every experiment for validation of staining strategies and to monitor technical variability (Supplementary Figure 1, 16 independent experiments).

We detected a sharp rise in proportion of DCs (CD11c^+^I-A/E^+^, % of live lin-, Figure 1D) in decidua of early gestational age (GD6-8, *p* < 0.0001), with subsequent decline starting in middle gestational age (GD12-14, *p* < 0.0005) with further decline in late gestational age (GD16-18, *p* < 0.0001). In contrast, on the fetal side of the interface (placenta), DCs were entirely excluded during middle (*p* < 0.0001) and late pregnancy (*p* < 0.0001) compared to their proportion in the embryo (GD6-8). In addition, compared with the virgin uterus, the DCs also increase in the embryo (GD6-8; *p* < 0.0005). Note that the immune cells isolated from the placenta and embryos are fetal in origin and reflect development of fetal nascent immune system. Taken together, DCs are dramatically expanded in early pregnancy decidua but entirely excluded from the placental compartment.

As CD11c+ I-A/I-E^+^ compartment includes both myeloid and lymphoid DCs, we investigated gestational subset dynamics: (a) Myeloid DCs, defined as lin1-CD11c^+^I-A/I-E^+^B220^neg^CD11b^+^CD8α^neg^ (Figures 1C,E); and (b) Lymphoid CD8α+ DCs, defined as lin1-CD11c^+^I-A/I-E^+^B220^neg^CD11b^neg^CD8α^+^ (Figures 1C,F). Interestingly, myeloid DCs predominate in the decidua, while CD8α+ DCs were a proportionally small subset, especially in light of their considerable proportion in non-gestational control tissues (Figures 1E,F, Supplementary Figure 1B). Myeloid DCs, in the decidua, showed a bimodal pattern, with early increase (*p* < 0.05), mid-pregnancy dip (*p* < 0.05) and late recovery of myeloid subsets (*p* < 0.005, Figure 1E). In the fetal compartment, early myeloid DCs are identified in the embryo (GD6-8). Finally, CD8α DCs were low and not significantly different across gestational reproductive tissues tested (Figure 1F).

### Plasmacytoid DCs are excluded from decidua

**Figure 2:**
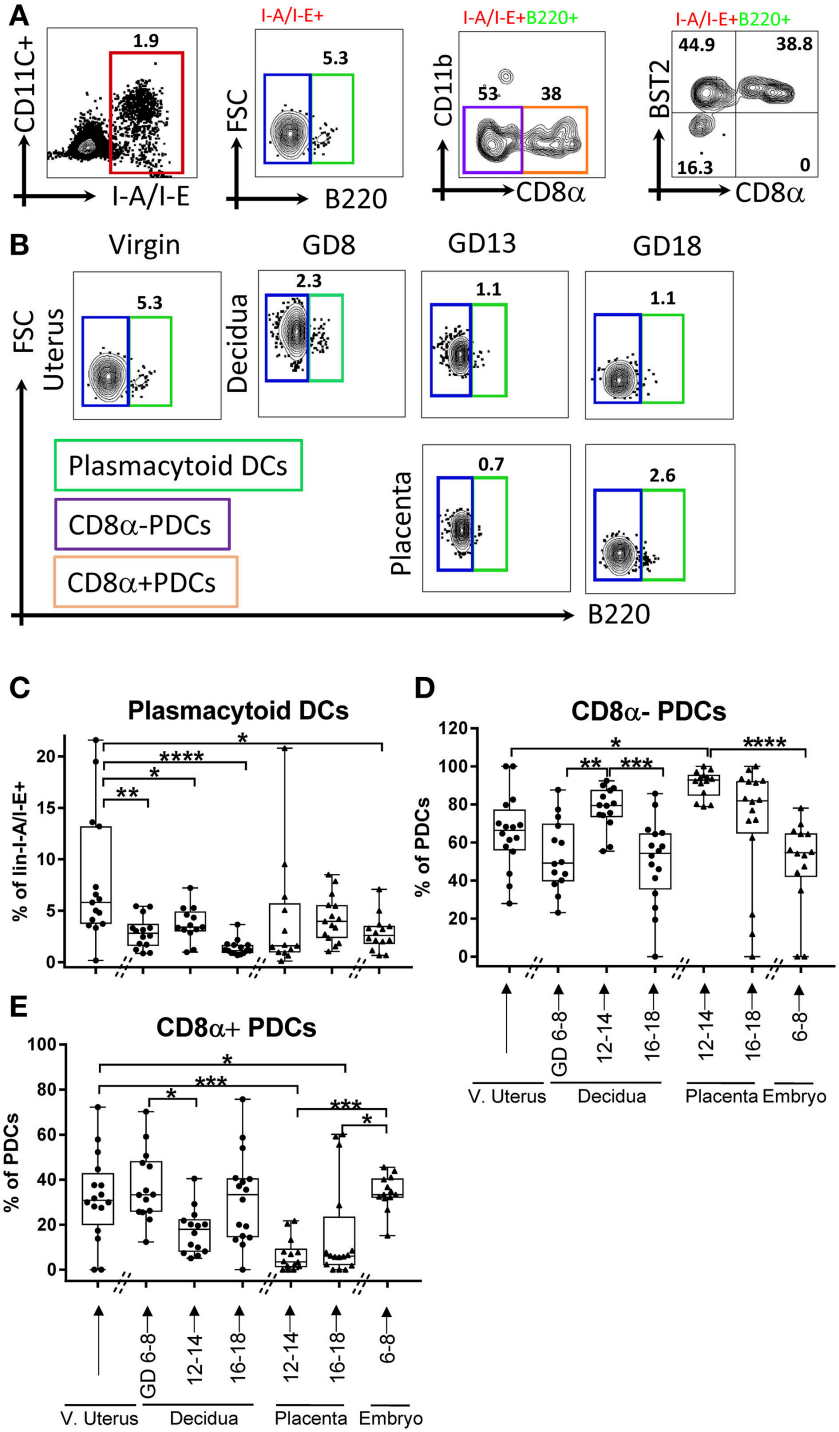
Flow cytometry gating strategy and dynamics of plasmacytoid dendritic cells (PDCs) in the murine uterus, decidua, placenta, and embryo. **(A)** PDCs, defined as live and lineage negative (CD3^neg^NK1.1^neg^CD19^neg^L-Y-6G^neg^) I-A/I-E^+^B220^+^, and its subsets CD8α+/– PDCs, were identified in the murine decidua. **(B)** Gating of lin1-I-A/I-E+B220+/– in murine virgin uterus, decidua, placenta, and embryo across the gestational age, upper panel showed decidua, lower panel showed as placenta. **(C)** Proportions of PDCs out of lin1-I-A/I-E+ in murine virgin uterus, decidua placenta, and embryo across gestational age. **(D)** Proportions of CD8α-PDCs out of PDCs in murine virgin uterus, decidua, placenta and embryo across gestational age. **(E)** Proportions of CD8α+PDCs out of PDCs in murine virgin uterus, decidua, placenta, and embryo across gestational age. *n* = 13–16 (16 independent experiments), the exact number of animals was summarized in Supplementary Table 3. The data of virgin uterus were compared with decidua group or placenta (include embryo) group, respectively. Statistical analysis was performed using ANOVA followed by *post-hoc* Tukey analysis. *p < 0.05, **p < 0.005, ***p < 0.0005, ****p < 0.0001.

Gestational dynamics of plasmacytoid DCs (PDCs, lin1-I-A/I-E^+^B220^+^CD11b^neg^, Figures 2A,B), and their subsets based on CD8α: CD8α^+^ and CD8α^neg^ PDCs (Figure 2A) were also analyzed PDCs were largely BST2 positive (Figure 2A, right panel). Interestingly, only a small minority of decidual DCs were PDCs (Figures 2A,C), and they were much lower in the decidua when compared with virgin uterus (Figure 2C, *p* < 0.05–0.0001). Within those present, CD8α^neg^ population predominated and unlike lymphoid PDCs, showed distinct gestational dynamics (Figures 2D,E). In the maternal compartment, early gestational age was characterized by lower CD8α^neg^ PDC (Figure 2D) proportion (*p* < 0.005), with mid-pregnancy recovery (*p* < 0.005), and drop again at late gestational age (*p* < 0.0005), showing the opposite pattern of CD8α^+^PDCs (Figure 2E). In the fetal compartment, CD8α^neg^ population predominated amongst PDCs, with higher proportion in placenta of mid-gestational age than embryo (*p* < 0.0001).

### Gestational redistribution of T cell subsets

**Figure 3:**
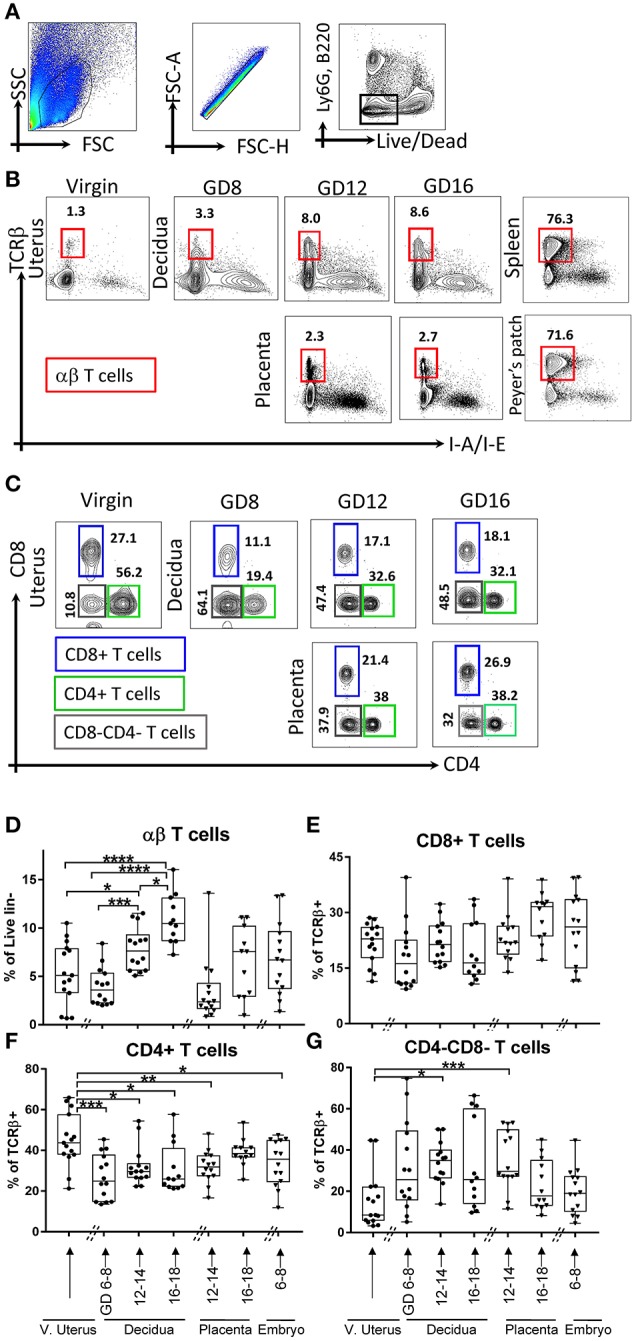
Gating strategy and dynamics of αβT cells. **(A,B)** αβT cells, defined as lin2-[Ly6G^neg^B220^neg^I-A/I-E^neg^ (TCRβ^+^)]. **(B)** Gating strategy of αβT cells in murine virgin uterus, decidua and placenta across the gestational age, upper panel showed decidua, lower panel showed as placenta, spleen, and Peyer's patches used as controls. **(C)** Gating strategy of T cells subsets, CD4+ T cells, CD8+ T cells, CD4-CD8-T cells. **(D)** Proportions of αβT cells out of live lin2-population. **(E–G)** Proportions of CD8+ T cells out of αβT cells), proportions of CD4+ T cells out of αβT cells, proportions of CD4-CD8-T cells out of αβT cells in murine virgin uterus, decidua, placenta, and embryo across gestational age. *n* = 11–15 (15 independent experiments), the exact number of animals was summarized in Supplementary Table 3. The data of virgin uterus were compared with decidua group or placenta (include embryo) group, respectively. Statistical analysis was performed using ANOVA followed by *post-hoc* Tukey analysis. *p < 0.05, **p < 0.005, ***p < 0.0005, ****p < 0.0001.

Dominant function of DCs is antigen presentation to T cells and instruct their subset differentiation (2). As we found complex DC dynamics during pregnancy, we hypothesized that T cell and their subsets also follow a characteristic dynamic across gestation, even in a syngeneic context. Therefore, we validated a 16-marker αβT cells panel (Table 1) to examine T cell subsets composition at the maternal-fetal interface across gestation (Figures 3A–C). We monitored T cells overall (Figures 3A,B, lin2-: Ly-6G^neg^B220^neg^I-A/I-E^neg^, TCRβ^+^cells) and examined subsets at maternal-fetal interface across gestation. Similar to DCs, T cells in control tissues including spleen, Peyer's patches, and thymus were determined in every analysis for validation of staining strategies and to monitor experimental variability (Supplementary Figure 3, 16 independent experiments).

In early gestation, we found a decrease in decidual αβT cells proportion compared to virgin uterus, followed by recovery and increase toward the end of pregnancy (*p* < 0.05–0.0001, depending on time point, Figure 3D), and the αβT cells proportion of middle and late pregnancy in decidua are higher than that of virgin uterus. Overall T cell dynamics was opposite that of CD11c+ I-A/I-E+ DCs in decidua (Figures 1D, 3D).

Major lineages of αβT cells across pregnancy, including a) CD4+ (Figure 3C); b) CD8+ (Figure 3C); c) CD4-CD8-T cells (Figure 3C) were further studied. We found that decidual and placental CD8+ T cells proportion was stable (Figure 3E). Interestingly, CD4+T cells were lower in decidua throughout the gestation and in fetal tissues (early embryo, mid-gestation placenta) compared to that of virgin uterus (*p* < 0.05–0.005, Figure 3F). Finally, we were surprised to find that CD4-CD8-T cells were dramatically increased in decidua (*p* < 0.05) and placenta (*p* < 0.0005) of middle pregnancy (GD12-14) compared with maternal uterus (Figure 3G) and all control tissues (Supplementary Figure 3A).

**Figure 4:**
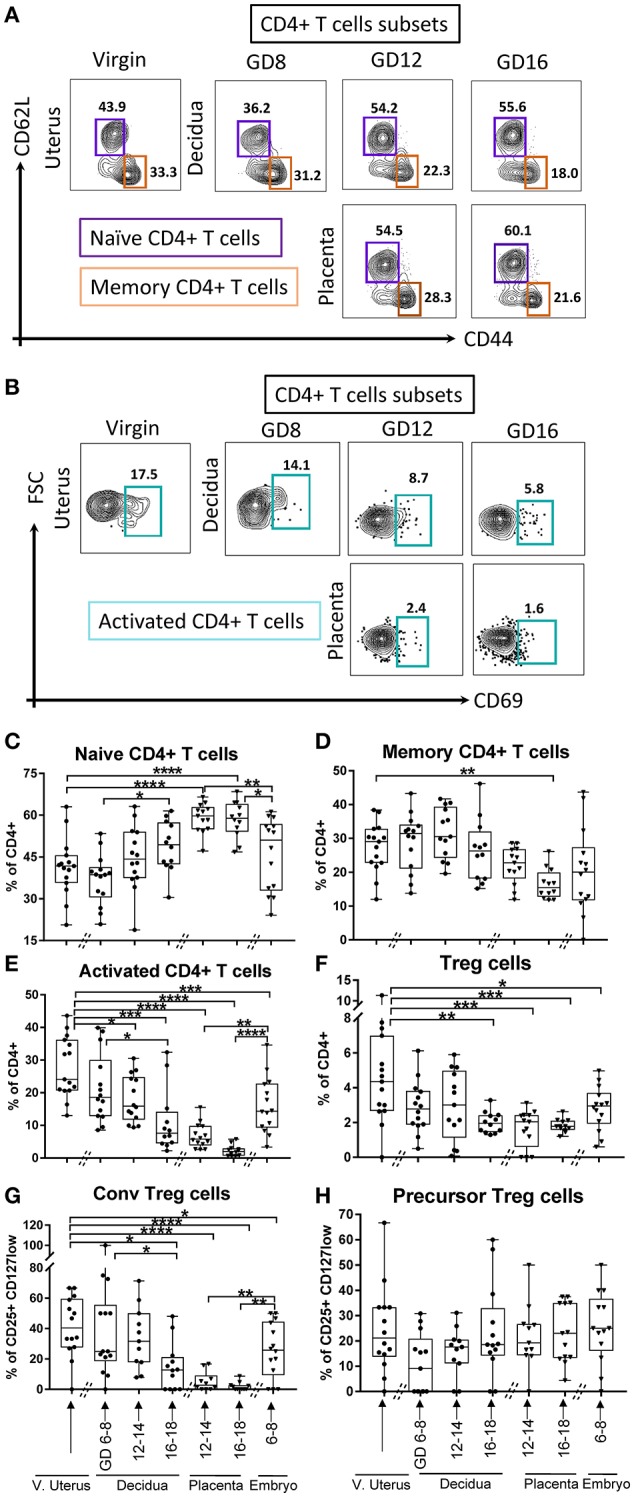
Gating strategy and dynamics of CD4+ T cells subsets. **(A)** Gating strategy of Naïve CD4+ T cells, defined as lin2- (Ly6G^neg^B220^neg^I-A/I-E^neg^) TCRβ^+^ CD4^+^CD62L^+^CD44^neg^, and memory CD4+ T cells, defined as lin2- (Ly6G^neg^B220^neg^I-A/I-E^neg^) TCRβ^+^CD4^+^CD62L^neg^CD44^+^. **(B)** Gating strategy of activated CD4+ T cells, defined as lin2-(Ly6G^neg^B220^neg^I-A/I-E^neg^) TCRβ^+^CD4^+^CD69^+^. **(C–E)** Proportions of Naïve CD4+ T cells, memory CD4+ T cells, and activated CD4+ T cells in murine virgin uterus, decidua, placenta, and embryo across gestational age. **(F–H)** Proportions of Tregs out of CD4+ T cells (lin2-TCRβ^+^CD4^+^CD25^+^CD127^low^) and the proportions of its subsets, conventional Tregs(CD69^+^CD62L^neg^), and precursor Tregs (CD69^neg^CD62L^+^) out of Tregs in murine virgin uterus, decidua, placenta, and embryo across gestational age. *n* = 11–15 (15 independent experiments), the exact number of animals was summarized in Supplementary Table 3. The data of virgin uterus were compared with decidua group or placenta (include embryo) group, respectively. Statistical analysis was performed using ANOVA followed by *post-hoc* Tukey analysis. *p < 0.05, **p < 0.005, ***p < 0.0005, ****p < 0.0001.

Next, we examined the dynamics of CD4+ lineage subsets. We differentiated naïve (CD62L^+^CD44^neg^), memory (CD62L^neg^CD44^+^), and activated (CD69^+^) CD4+ αβT cells (Figures 4A,B). In the decidua, we detected an increase of naïve CD4+ T cells proportion in late compared to early pregnancy (Figure 4C, *p* < 0.05); while activated CD4+ T cells proportion was lower in middle and late pregnancy (Figure 4E, *p* < 0.05 and *p* < 0.0005, respectively), compared to virgin uterus. Amongst fetal cells, naïve CD4+ T cells predominated in placenta (Figure 4C, *p* < 0.0005), while activated CD4+ T cells were proportionally low/excluded (Figure 4E, *p* < 0.005). Memory CD4+ T cells were stable across gestation in the decidua, but low in placenta of late pregnancy (Figure 4D, *p* < 0.005). Decidual CD4+ cells with a “regulatory” phenotype (Tregs, CD127^low^CD25^+^CD4^+^, Supplementary Figure 4), were low at late pregnancy in decidual and across gestation in placental locations (Figure 4F). Tregs phenotypes also showed distinct dynamics, with conventional Tregs decreased in late pregnancy decidua and very low in the placenta (CD62L^neg^CD69^+^, Supplementary Figure 4, Figure 4G, *p* < 0.05–0.005). On the other hand, precursor-type Tregs proportion (CD62L^+^CD69^neg^, Supplementary Figure 4) was stable across tissues and time (Figure 4H).

**Figure 5:**
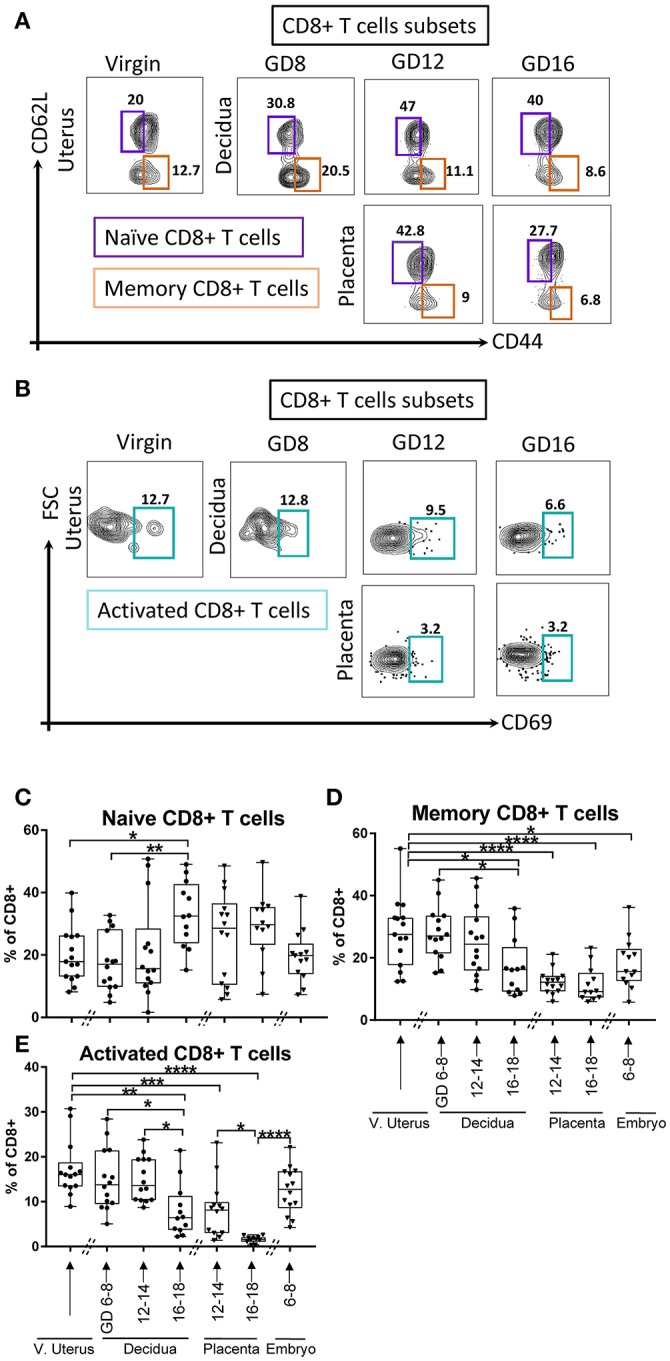
Gating strategy and dynamic change of CD8+ T cells subsets. **(A)** Gating strategy of Naïve CD8+ T cells, defined as lin2- (Ly6G^neg^B220^neg^I-A/I-E^neg^) TCRβ^+^ CD8^+^CD62L^+^CD44^neg^, and memory CD8+ T cells, defined as lin2-(Ly6G^neg^B220^neg^I-A/I-E^neg^) TCRβ^+^ CD8^+^CD62L^neg^CD44^+^. **(B)** Gating strategy of activated CD8+ T cells, defined as lin2-(Ly6G^neg^B220^neg^I-A/I-E^neg^) TCRβ^+^CD8^+^CD69^+^. **(C–E)** Proportions of Naïve CD8+ T cells, memory CD8+ T cells, and activated CD8+ T cells in murine virgin uterus, decidua, placenta, and embryo across gestational age. *n* = 11–15 (15 independent experiments), the exact number of animals was summarized in Supplementary Table 3. The data of virgin uterus were compared with decidua group or placenta (include embryo) group, respectively. Statistical analysis was performed using ANOVA followed by *post-hoc* Tukey analysis. *p < 0.05, **p < 0.005, ***p < 0.0005, ****p < 0.0001.

Similarly, CD8+ T cells lineage subsets with naïve (CD62L^+^CD44^neg^, Figure 5A), memory (CD62L^neg^CD44^+^, Figure 5A), and activated phenotypes (CD69^+^, Figure 5B) were investigated. In the maternal compartment, the naïve CD8+ T cells were stable in early and middle pregnancy compared with virgin uterus, but expanded in late gestational age (Figure 5C, *p* < 0.05, *p* < 0.005, virgin uterus and decidua in early gestational age, respectively). Corresponding decline in memory (Figure 5D, *p* < 0.05) and activated (Figure 5E, *p* < 0.0001) CD8+ proportion was seen at late gestational age. In the fetal compartment, the proportion of memory CD8+ T cells was lower in early embryo (Figure 5D, *p* < 0.05), and placenta excluded CD8+ cells with a memory (Figure 5D, *p* < 0.0001) and activated (Figure 5E, *p* < 0.0001) phenotype.

### Interface DC- and T-subsets visualized by dimensionality reduction and machine learning

Given the complexity of DCs and T cells compartments, and difficulty in assigning subset phenotypes by 2-dimensional gating section through 18-dimensional parameter space, we employed dimensionality reduction and machine learning (DensVM) methods to parse the full dataset.

**Figure 6:**
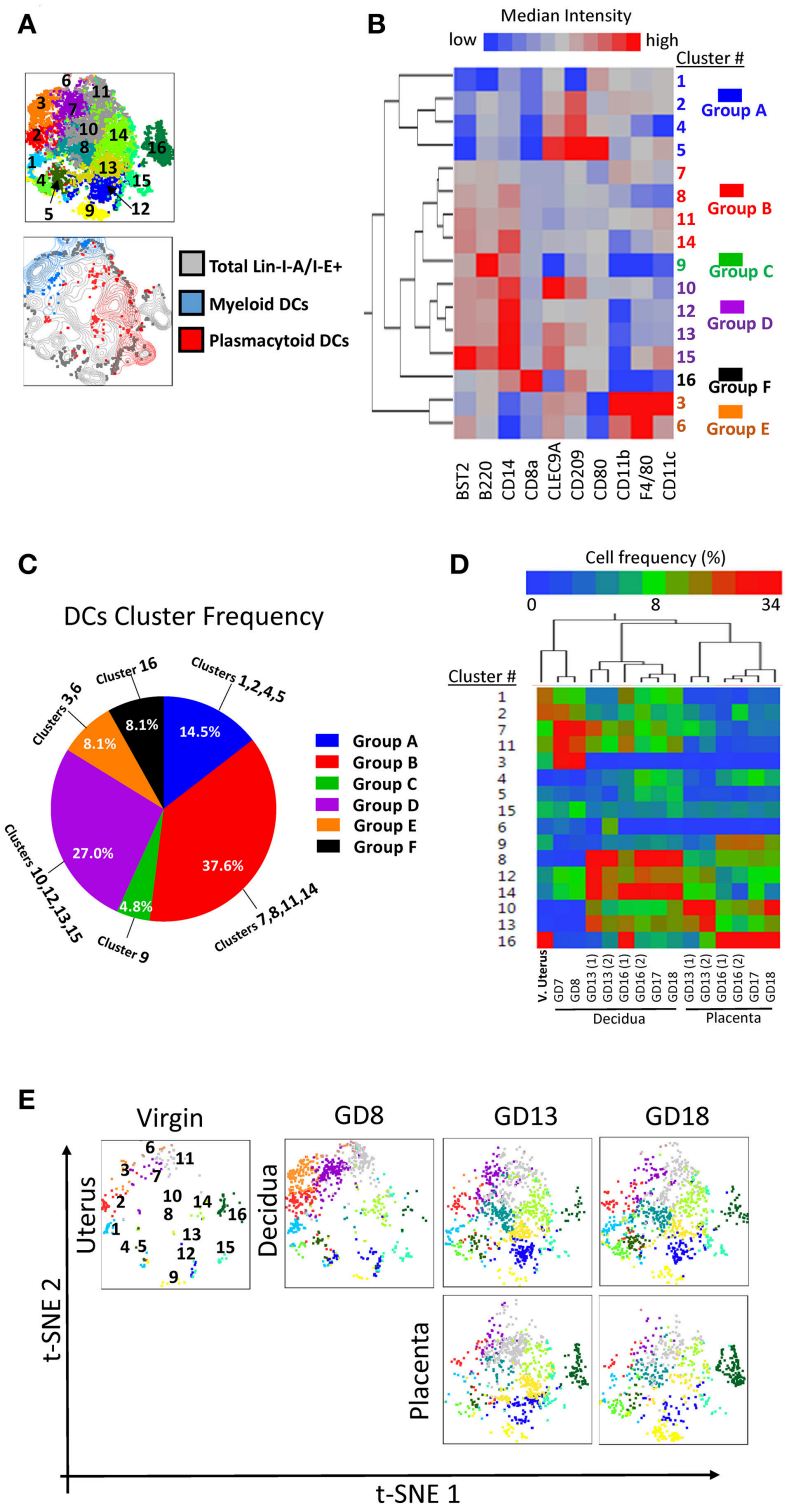
Visualization of dendritic cell diversity. To avoid the cross-batch effect, we used a single experiment including 15 samples of virgin uterus, decidua and placenta across the whole murine pregnancy. **(A)** t-SNE map generated from pre-gated lin1-I-A/I-E+ cells from murine virgin uterus, decidua and placenta across gestational age data sets (top) and manually gated subsets overlaid onto total lin1-I-A/I-E+ cells (bottom). **(B)** Hierarchical clustering of median surface marker expression levels of clusters identified by DensVM. Total of 16 clusters were categorized in 6 groups, group A–F. **(C)** Pie chart of different group proportions of total DC population. **(D)** Hierarchical clustering of cluster frequency within lin1-I-A/I-E+ from murine virgin uterus, decidua and placenta across gestational age. **(E)** Separate visualization of cluster in different murine tissues across gestation using t-SNE map generated from the merged data set.

First, we examined the lin1-I-A/E+ compartment from coded/concatenated data of a single experiment that included 15 samples (virgin uterus, 8 decidual and 6 placenta specimens) covering all 3 gestational ages. Data was visualized on a 2D-map by t-distributed stochastic neighbor embedding (t-SNE) (26, 27) and visually partitioned into 16 clusters by support vector machine learning-aided analysis (DensVM, Figure 6A, top). To validate t-SNE/DensVM-derived cellular identity, myeloid DCs and plasmacytoid DCs were manually gated and overlaid onto the t-SNE map. As expected, myeloid DCs and plasmacytoid DCs were located at separate regions of the map and were marked as different clusters by the densVM algorithm (Figure 6A, bottom). A heatmap of the median fluorescence intensity (MFI) for every marker analyzed was generated to determine the phenotype of identified clusters (Figure 6B). Similarity mapping (hierarchical) suggested 6 broad categories of cells (Figures 6B,C) that did not always follow “classical” DC phenotypes (Supplementary Table 1). For example, group D, had a difficult to ascertain phenotype, most reminiscent of CD14+monocyte/macrophages. The groups A, B, C, D, E, F consist of 14.5, 37.6, 4.8, 27.0, 8.1, 8.1% of overall I-A/E+ cells (Figure 6C). Based on the cluster markers profile and matched manual characterization of DC subsets, cluster 3 and 6 (group E) were identified as myeloid DCs and cluster 14 and 15 were identified as plasmacytoid DCs (Table 2).

**Table 2:** Presumed classification of I-A/I-E^+^ clusters (Figure 6B).

**Cluster**	**Phenotype**	**Presumed name**
1	BST2^−^ B220^−^ CD14^−^ CD8a^−^ CLEC9A^INTER^ CD209^−^ CD80^+^ CD11b^INTER^ F4/80^−^ CD11c^INTER^	Undefined
2	BST2^−^ B220^INTER^ CD14^−^ CD8a^−^ CLEC9A^+^ CD209^+^ CD80^−^ CD11b^+^ F4/80^+^ CD11c^INTER^	Undefined
3	BST2^−^ B220^INTER^ CD14^−^ CD8a^−^ CLEC9A^+^ CD209^+^ CD80^−^ CD11b^+^ F4/80^+^ CD11c^+^	Myeloid DCs
4	BST2^−^ B220^INTER^ CD14^−^ CD8a^−^ CLEC9A^+^ CD209^+^ CD80^INTER^ CD11b^INTER^ F4/80^−^ CD11c^−^	Undefined
5	BST2^−^ B220^−^ CD14^−^ CD8a^−^ CLEC9A^+^ CD209^+^ CD80^+^ CD11b^−^ F4/80^INTER^ CD11c^+^	Undefined
6	BST2^+^ B220^INTER^ CD14^−^ CD8a^−^ CLEC9A^+^ CD209^INTER^ CD80^−^ CD11b^+^ F4/80^+^ CD11c^+^	Myeloid DCs
7	BST2^+^ B220^+^ CD14^INTER^ CD8a^−^ CLEC9A^−^ CD209^INTER^ CD80^−^ CD11b^+^ F4/80^+^ CD11c^−^	Undefined
8	BST2^+^ B220^+^ CD14^+^ CD8a^−^ CLEC9A^−^ CD209^INTER^ CD80^INTER^ CD11b^−^ F4/80^−^ CD11c^−^	Undefined
9	BST2^+^ B220^+^ CD14^+^ CD8a^−^ CLEC9A^−^ CD209^−^ CD80^INTER^ CD11b^−^ F4/80^−^ CD11c^−^	Undefined
10	BST2^+^ B220^+^ CD14^+^ CD8a^−^ CLEC9A^+^ CD209^+^ CD80^INTER^ CD11b^INTER^ F4/80^INTER^ CD11c^−^	Undefined
11	BST2^+^ B220^+^ CD14^+^ CD8a^−^ CLEC9A^−^ CD209^−^ CD80^−^ CD11b^INTER^ F4/80^+^ CD11c^+^	Undefined
12	BST2^+^ B220^+^ CD14^+^ CD8a^−^ CLEC9A^INTER^ CD209^INTER^ CD80^INTER^ CD11b^−^ F4/80^−^ CD11c^−^	Undefined
13	BST2^+^ B220^+^ CD14^+^ CD8a^−^ CLEC9A^+^ CD209^+^ CD80^INTER^ CD11b^−^ F4/80^−^ CD11c^−^	Undefined
14	BST2^+^ B220^+^ CD14^+^ CD8a^−^ CLEC9A^−^ CD209^INTER^ CD80^INTER^ CD11b^−^ F4/80^−^ CD11c^+^	PDCs
15	BST2^+^ B220^+^ CD14^+^ CD8a^−^ CLEC9A^+^ CD209^INTER^ CD80^INTER^ CD11b^−^ F4/80^INTER^ CD11c^+^	PDCs
16	BST2^−^ B220^INTER^ CD14^+^ CD8a^+^ CLEC9A^−^ CD209^+^ CD80^INTER^ CD11b^−^ F4/80^−^ CD11c^−^	Undefined

When we investigated each cluster in different tissues across pregnancy, we found that the hierarchical two-way clustering grouped tissues together (i.e., all decidual specimens and all placental specimens were separate, Figure 6D). Thus, cluster composition of individual tissue formed an “immune signature,” identifying the tissue and pregnancy timing, similar to the previously demonstrated peripheral blood “immune clock of pregnancy” (28). t-SNE map also allowed sequential visualization of all the clusters in individual specimens across pregnancy (Figure 6E).

**Figure 7:**
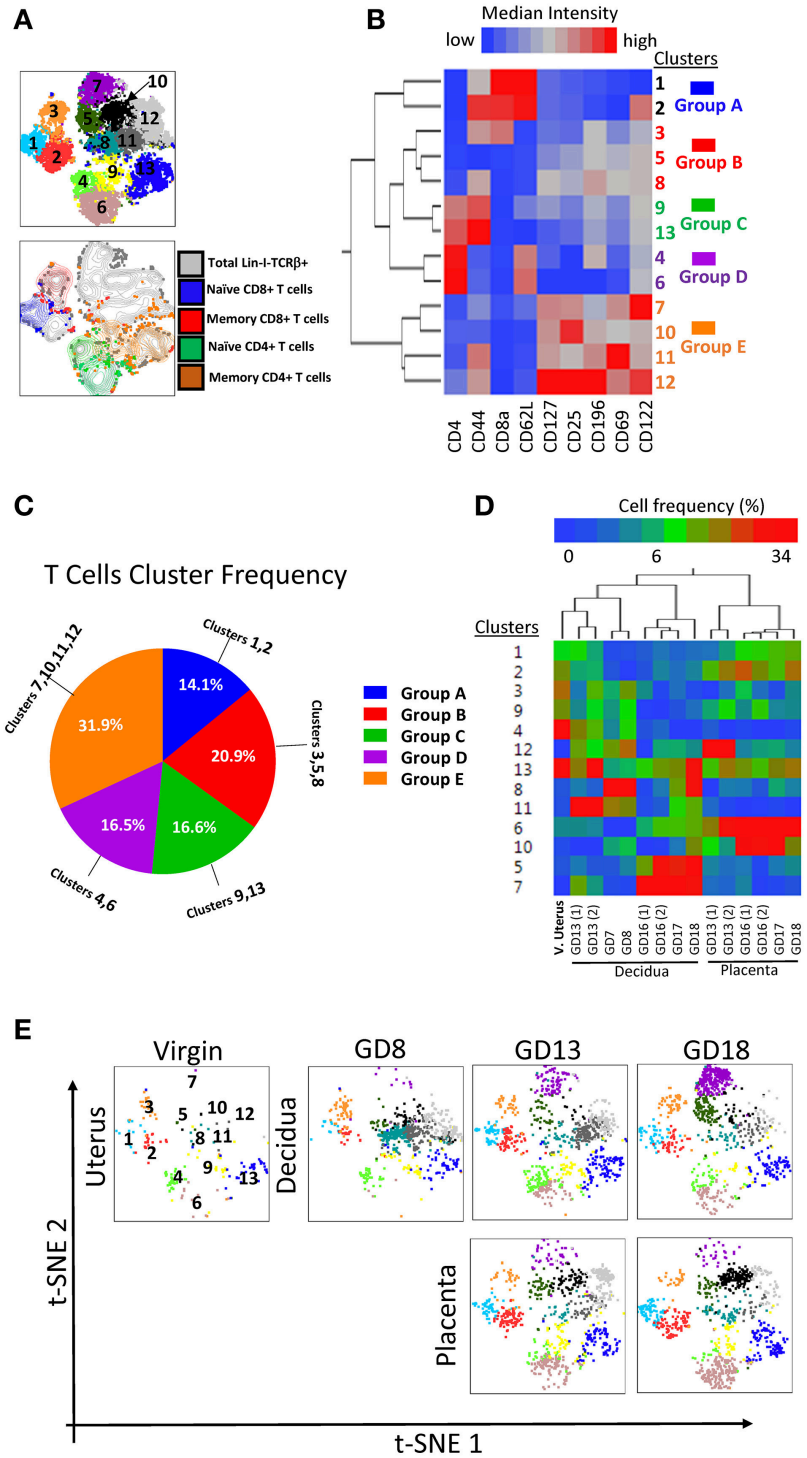
Visualization of T cell diversity. **(A)** t-SNE map generated from pre-gated lin2-TCRβ+ cells from murine virgin uterus, decidua and placenta across gestational age data sets (top) and manually gated subsets overlaid onto total lin2-TCRβ+ cells (bottom). **(B)** Hierarchical clustering of median surface marker expression levels of clusters identified by DensVM. Total 13 clusters were categorized in 5 groups, group A–E **(C)** Pie chart of different group proportions of total DC population. **(D)** Hierarchical clustering of cluster frequency within lin2-TCRβ+ from murine virgin uterus, decidua and placenta across gestational age. **(E)** Separate visualization of cluster in different murine tissues across gestation using t-SNE map generated from the merged data set.

Similarly, we applied the t-SNE/densVM pipeline to the analysis of maternal-fetal interface T cells. DensVM identified 13 clusters within αβT cells (lin2-TCRβ+) on the t-SNE map (Figure 7A, top). Manually gated CD8+ (Naïve and memory) and CD4+ (Naïve and memory) T cells were located at different region on the t-SNE map, again demonstrating the utility of the automated/unbiased approach (Figure 7A, bottom). A heatmap of the MFI for every marker analyzed within each cluster was generated to assess phenotypes (Figure 7B). These cluster were divided into 5 broad groups based on the cluster similarity (Figures 7B,C, Supplementary Table 2). Thus subdivided, groups A, B, C, D, E consisted of 14.1, 20.9, 16.6, 16.5, 31.9%, respectively (Figure 7C, Table 3).

**Table 3:** Presumed classification of TCRβ^+^ clusters (Figure 7B).

**Cluster**	**Phenotype**	**Presumed name**
1	CD4^−^ CD44^INTER^ CD8a^+^ CD62L^+^ CD127^−^ CD25^−^ CD196^−^ CD69^−^ CD122^−^	Naïve CD8+ T cells
2	CD4^−^ CD44^+^ CD8a^+^ CD62L^+^ CD127^−^ CD25^−^ CD196^−^ CD69^−^ CD122^+^	Undefined
3	CD4^−^ CD44^+^ CD8a^+^ CD62L^−^ CD127^−^ CD25^−^ CD196^INTER^ CD69^−^ CD122^−^	Memory CD8+ T cells
4	CD4^+^ CD44^−^ CD8a^−^ CD62L^+^ CD127^−^ CD25^−^ CD196^INTER^ CD69^−^ CD122^−^	Naïve CD4+ T cells
5	CD4^−^ CD44^−^ CD8a^−^ CD62L^−^ CD127^−^ CD25^−^ CD196^INTER^ CD69^−^ CD122^INTER^	Undefined
6	CD4^+^ CD44^−^ CD8a^−^ CD62L^+^ CD127^−^ CD25^−^ CD196^−^ CD69^−^ CD122^INTER^	Naïve CD4+ T cells
7	CD4^−^ CD44^−^ CD8a^−^ CD62L^−^ CD127^+^ CD25^+^ CD196^−^ CD69^+^ CD122^+^	Double negative T cells
8	CD4^−^ CD44^+^ CD8a^−^ CD62L^−^ CD127^INTER^ CD25^−^ CD196^+^ CD69^INTER^ CD122^INTER^	Undefined
9	CD4^+^ CD44^+^ CD8a^−^ CD62L^−^ CD127^−^ CD25^−^ CD196^−^ CD69^−^ CD122^INTER^	Memory CD4+ T cells
10	CD4^−^ CD44^−^ CD8a^−^ CD62L^−^ CD127^+^ CD25^+^ CD196^INTER^ CD69^INTER^ CD122^INTER^	Double negative T cells
11	CD4^−^ CD44^+^ CD8a^−^ CD62L^−^ CD127^+^ CD25^INTER^ CD196^+^ CD69^+^ CD122^+^	Double negative T cells
12	CD4^−^ CD44^+^ CD8a^−^ CD62L^−^ CD127^+^ CD25^+^ CD196^+^ CD69^+^ CD122^+^	Double negative T cells
13	CD4^+^ CD44^+^ CD8a^−^ CD62L^−^ CD127^−^ CD25^−^ CD196^−^ CD69^−^ CD122^INTER^	Memory CD4+ T cells

Matching the cluster markers profile and manual characterization of T cell subsets, we identified cluster 1 (Group A) as naïve CD8+ T cells, cluster 3 (group B) as memory CD8+ T cells, clusters 4 and 6 (group D) as naïve CD4+ T cells and clusters 9 and 13 (group C) as memory CD4+ T cells. Interestingly, in this analysis, similar to the classical CD4/CD8 gating, we find a group of broad and populous clusters that are CD4-CD8-, prevalent in mid-late pregnancy, and with a broadly distinct phenotype compared with groups A-D (group E, Figures 7C,D). Similar to DCs, T cells distribution also exhibited a tissue and time signature specific to pregnancy (Figure 7D). These were recapitulated on t-SNE maps of individual samples (Figure 7E).

Taken together, the use of tSNE/densVM pipeline allowed us to visualize the complex manual analysis in a simple and concise fashion. Furthermore, clustering analysis revealed unexpected, broad differences in the phenotype of CD4-CD8-T cells compared with conventional CD4+ and CD8+ T cells in the decidua. Finally, a tissue- and time-dependent signature of gestational specimens is evident.

## Discussion

Adaptive immune cells at the maternal-fetal interface are essential immune mediators necessary for a successful pregnancy. The composition and function of interface immune cells changes throughout the pregnancy to adjust to different pregnancy challenges. Consequently, dysregulation of adaptive immune composition and function is highly associated with pregnancy complications (preeclampsia, intrauterine growth restriction-IUGR, etc.) (1, 16). Introduction of highly polychromatic flow cytometry and computational analysis methods allowed us to perform a normative re-evaluation of adaptive immune axis (T-DC) gestational dynamics in the syngeneic mouse model, demonstrating applicability in this complex tissue. It is important to note that a variety of genetic (maternal/fetal major and minor histocompatibility antigens), environmental (systemic and local microbiomes) and hormonal influences have a potential role in DC/T cell dynamics. Here we validate the approach to increased data density and computational analysis pipeline employed by highly polychromatic flow cytometry and with conventional analysis and machine learning interpretation of immune cells in gestational compartments (decidua, placenta). While it is not faithful to physiological pregnancy in humans, or wild non-laboratory mice, it provides a methodological guide to high-dimensional gestational analysis. Furthermore, as this study focuses on establishing the map of immune composition, our interpretation of potential functional significance is necessarily speculative and meant to inform hypothesis-testing.

First, we find a dramatic CD11c+I-A/I-E+ DCs dynamic—massive early expansion, followed by gradual proportional decrease toward delivery. Early pregnancy expansion suggests that DCs are important in the early events of embryo implantation and decidual remodeling. Blois et al. (29), first suggested that proportion of CD11c+ cells isolated from mouse uterus (including uterus, decidua and myometrium in the study) begins to increase at gestation day 5.5 and reaches a peak at day 8.5 (early pregnancy), followed by decrease toward the middle and late gestation (days 10.5, 13.5, 15.5, 17.5). It is still unclear if the expansion of DCs in early pregnancy is due to active import from the peripheral circulation, from the proliferation of resident immune cells, or combination of both. DCs are trapped in the decidua during pregnancy in mice (12), consistent with both possibilities, as expansion may be the result of proliferation of resident DCs or active trapping of cells that traffic through decidua. Also, more importantly, CD11c+I-A/I-E+DCs are primarily found in the decidual tissue, but not in the placental tissue suggesting a specialized decidual function. Previously, Fainaru et al. investigated the DCs (CD45^+^CD11c^+^MHCII^+^) exclusively in the placental compartment, and found that proportion of mouse placental DCs/CD45^+^ cells is low in middle pregnancy (days 12, 15), with some accumulation toward the end of pregnancy (30), although as they did not investigate decidua, the major difference between decidua and placenta was not apparent in this work.

DCs subsets, which show differential pattern of surface marker expression (Supplementary Figure 2), have distinct functions in immune physiology. Lymphoid CD8α+DCs induce the Th1 cytokines like interferon gamma and interleukin (IL)-2 while myeloid CD8α-DCs induce Th2 cytokines IL-4 and IL-10 (31, 32). First, we find that myeloid DCs are the dominant population (Figures 1E,F), which is consistent with prior work (29). Second, the dynamics of myeloid DCs and lymphoid DCs were distinct, with the proportion of myeloid DCs increased in early pregnancy while CD8α^+^ DCs showed the opposite trend in early pregnancy in decidua (Figures 1E,F), helping to explain the known Th2 bias of pregnancy. Third, while we found there was very few PDCs (I-A/I-E^+^B220^+^) in decidua, they were present in the placenta (Figure 2C). As CD11c+I-A/I-E+DCs were completely excluded from placenta (Figure 1D), PDC exclusion of CD11c+I-A/I-from decidua is likely functionally meaningful. Outside of the utero-placental compartment (mouse gestational para-aortic lymph nodes), conventional DCs (CD11c^+^) are reduced while PDCs (CD11c^−tolo^B220^+^PDCA-1^+^) increased from (E8.5) early to (E16.5) late pregnancy (33), possibly reflecting trapping of conventional DCs within the decidua and export of PDCs.

The overall αβT cell dynamics was opposite to that of CD11c+I-A/I-E+DCs in decidua (Figures 1D, 3D), suggesting the possible interaction of CD11c+ I-A/I-E+ DCs and αβT cells in the decidua during pregnancy. Early, and massive DC accumulation, may physically displace T cells in early pregnancy, alternatively, T cells population increase in late pregnancy could be in response to diminished DC inhibition or local priming. Consistent to our data in mice (Figure 3D), Vassiliadou and Bulmer also showed that T cells decreased in early pregnancy in human (34). Interestingly, in regard to the subset of DCs and T cells, we showed that the dynamics of PDCs (Figure 2C) and Tregs (Figure 4F) in the decidua share the same pattern, indicating the correlation between the specific subset of DCs and T cells that Tregs may be primed by PDCs (33).

CD4-CD8-T cells have been reported to play important roles in many tissue of different species. For example, αβTCR^+^CD3^+^CD4^−^CD8^−^ T cells have been shown to inhibit a variety of immune responses by directing killing of effector T cells in an antigen specific manner in both human and mice (35). Lung CD4-CD8-double negative T cells are primarily responsible for producing IL-17A and IFN-γ during respiratory murine infection with Francisella Tularensis live vaccine strain (36). It is interesting that we find accumulation of CD4-CD8-T cells in decidua and placenta. To our knowledge, this specific subpopulation of T cells has not been reported in the mouse decidua or placenta, although there is a study by Joansson and Lycke that detected αβTCR+CD4-CD8-T cells (CD3^+^αβTCR^int^CD4^−^CD8^−^B220^low^) as dominant lymphocytes in the mouse female genital tract (37). We are in the process of investigating this curious finding further.

Tregs play an essential role in maintaining pregnancy (38, 39), and prior studies showed that Tregs (CD4^+^CD25^high^) proportion is higher in decidua than periphery in early pregnancy in humans (38, 39). Our model is syngeneic and consistent with prior studies that CD4^+^CD25^+^ were not dramatically altered in pregnant mice (40), suggesting a primary role in control of alloresponse (41). We show that proportion of Tregs in syngeneic C57BL/6 pregnancy significantly declined at late gestation in decidua and was low in placenta consistent with the prior work using Foxp3 as the Tregs-defining marker (42).

Different subsets of T cells may be primed by different subsets of DCs, since myeloid but not lymphoid DCs cross-prime CTLs (CD8^+^) in mice (43). In decidua, our data did not demonstrate prominent change in CTL (CD8+T cells) or lymphoid (CD8α) DC frequency across gestation, however, their functional responsiveness was not investigated. Placenta, in contrast, demonstrated accumulation of CD8α T cells in late pregnancy, and a non-significant trend toward increase in late gestation lymphoid DCs, suggesting that CD8+ T cells at the fetal side may be primed by the lymphoid DCs although very few are present.

Our data demonstrates the divergence of proportion and dynamics of adaptive immune cells subsets in maternal decidua and fetal placenta. As noted, DCs predominately accumulate in the decidua while exclude in placenta, naïve but not active CD4+ T cells predominated in placenta, indicating the majority of nascent fetal CD4 lymphocytes have not encounter antigen/antigen-presenting cells (16). These findings were vividly demonstrated by the machine learning aided clustering of decidual and placental samples (Figures 6D, 7D). Finally, although we have kept the virgin mice during rearing in the same cage as the experimental animals, variability in this group is a limitation and to be expected as we have not explicitly tested the estrous stage of the isolated virgin uteri.

The high heterogeneity of DCs and T cells required high-dimensional flow cytometry with multiple markers to define the specific subsets as precisely as possible. Dimensionality reduction/machine learning algorithms promises the simplicity and standardization of high dimensional data in an unbiased fashion (44). Therefore, in addition to the manual analysis, we also employed operator-independent dimensionality reduction and machine learning algorithms for cellular subset identification and tracking their gestational dynamics to verify manual analysis and explore their future application in evaluating the maternal-fetal immunome. Computational methods not only verified our manual analysis such as that cluster 3 (identified as myeloid DCs, group E, Figure 6B) showed the same dynamics (Figure 6D) with myeloid DCs manual gating (Figure 1E), it also identified previous unknown subsets. Amongst MHC Class II expressing cells (I-A/I-E+), cluster 9 (Figure 6B) is lin-I-A/I-E^+^ BST2^+^B220^+^CD14^+^CD80^inter^CD8a^low^CD11b^neg^ F4/80^neg^CD11c^neg^, which does not match known systemic DC subsets, and may be an intermediate subset in development (pre-PDCs?). For TCRαβ+ population, the clusters 11, 12 (group E) are lin-TCRαβ^+^CD4^neg^CD8^neg^ CD62L^neg^CD44^+^CD127^+^ CD196^+^CD69^+^CD122^+^, and belongs to the unusual CD4-CD8-T cell population. Overall, little is known about double negative T cells in other tissues and even less in decidua (35). Furthermore, as demonstrated by the expression heatmap, CD4-CD8-T cells were very different in their overall expression of these non-TCR receptors, suggesting an alternate developmental/activation path.

Within the syngeneic pregnancy model, the complex and divergent patterns of multiple DC- and T-cell subsets reveals normative information on remodeling of DCs and T cells compartment at the maternal-fetal interface, with dramatic gestational dynamics implying their complex roles at different developmental times. Dimensionality reduction and DensVM cluster on t-SNE map allowed us to more clearly visualize the known and novel clusters in an unbiased and standardized fashion. These results provide a new normative framework for studies of pregnancy immunology in a reproducible fashion, with a focus on discovery of novel phenotypes.

## Author contributions

YL and AS designed the research. YL performed the majority of experiments with GL, YS, MC, SF, and NK. JV and PL assisted with machine learning analysis. YL and AS wrote the manuscript. AS supervised the project.

### Conflict of interest statement

The authors declare that the research was conducted in the absence of any commercial or financial relationships that could be construed as a potential conflict of interest.
