# Supplementary material for: Decidual-Placental Immune Landscape During Syngeneic Murine Pregnancy
Source: Front Immunol. 2018 Sep 19;9:2087. doi: 10.3389/fimmu.2018.02087 (PMC6156255; doi:10.3389/fimmu.2018.02087)
Supplement: Supplementary file 1 [file Data_Sheet_1.pdf]

Supplementary Material

Decidual-placental Immune Landscape During Syngeneic Murine Pregnancy

Yan Li[1], Gladys E. Lopez[1], Jessica Vazquez[1], Yan Sun[2,3], Melina Chavarria[1],

Payton N. Lindner[1], Samantha Fredrickson[1], Nathan Karst[1], Aleksandar K. Stanic[1,3,*]

Division of [1]Reproductive Sciences, [2]Reproductive Endocrinology and Infertility, Department of Obstetrics and Gynecology, University of Wisconsin-Madison, Madison, WI

[3]Reproductive Medicine Center, Fujian Provincial Maternity and Children's Hospital, Affiliated Hospital of Fujian Medical University.

***Corresponding authors:**

Aleksandar K. Stanic (stanickostic@wisc.edu)

202 S Park St,

Perinatal Research Laboratories,

Meriter Atrium B,

Madison, WI 53715

Email: stanickostic@wisc.edu

Supplementary Data

Phenotype differentiation among different subsets of DCs

To further distinguish to the phenotype of DCs subsets, we identified three subpopulations of DCs, plasmacytoid (lin1-I-A/I-$E^{+}B220^{+}$), CD8α+ (lin1-CD11c^{+}I-A/I-$E^{+}B220^{neg}CD11b^{neg}CD8\alpha^{+}$), myeloid DCs in uterine, decidua and placenta tissue across the pregnancy, as well as spleen, Peyer's patches and thymus (Supplementary Fig. 2). We found that in the spleen, BST2 expression is much higher in plasmacytoid DCs subpopulation, compared with CD8+ DCs and Myeloid DCs in spleen, Peyer's patches, thymus, conforming the variant phenotype of the three subpopulations within DCs.

Supplementary FIGURE 1

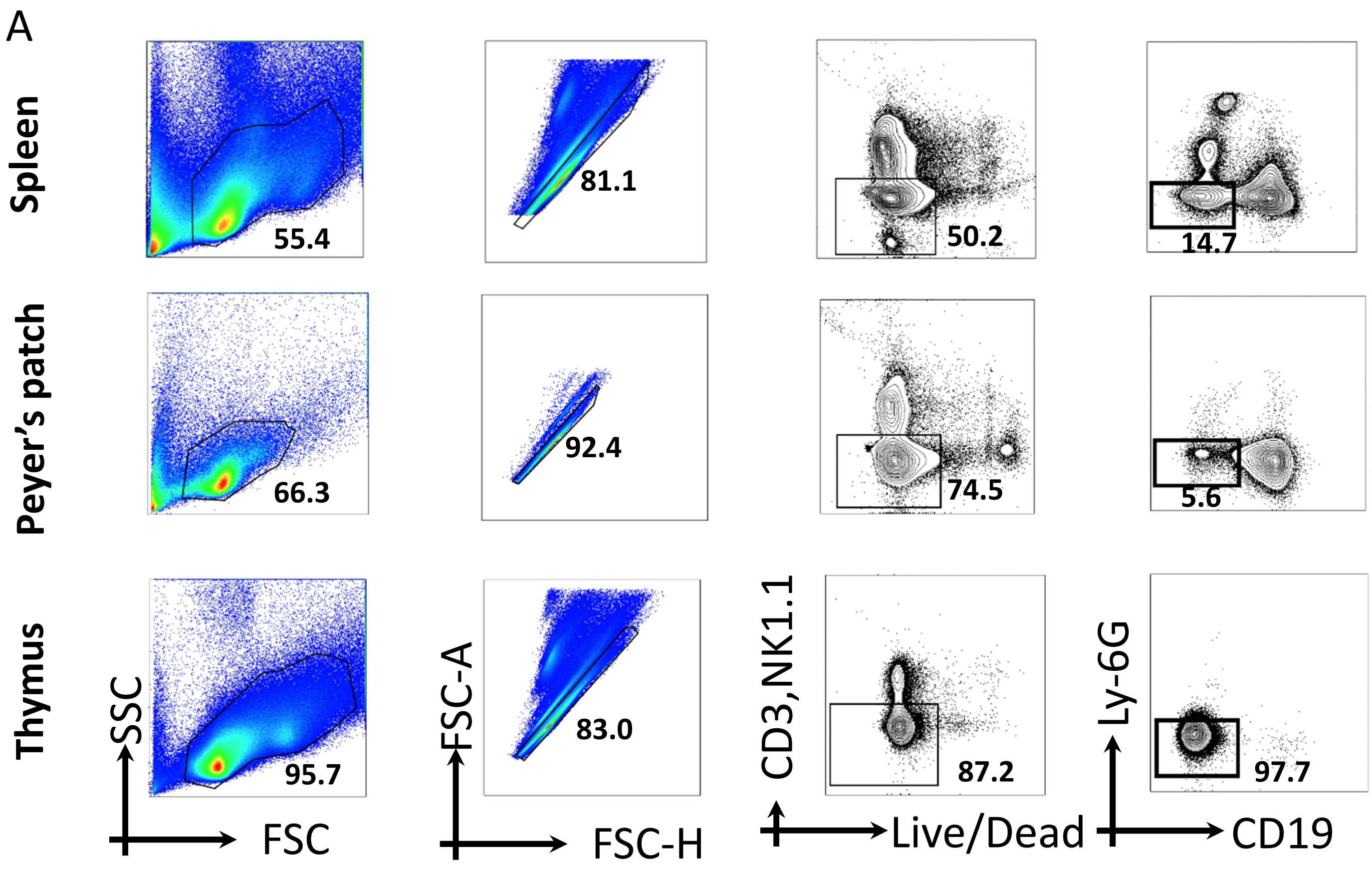

Supplementary FIGURE 1

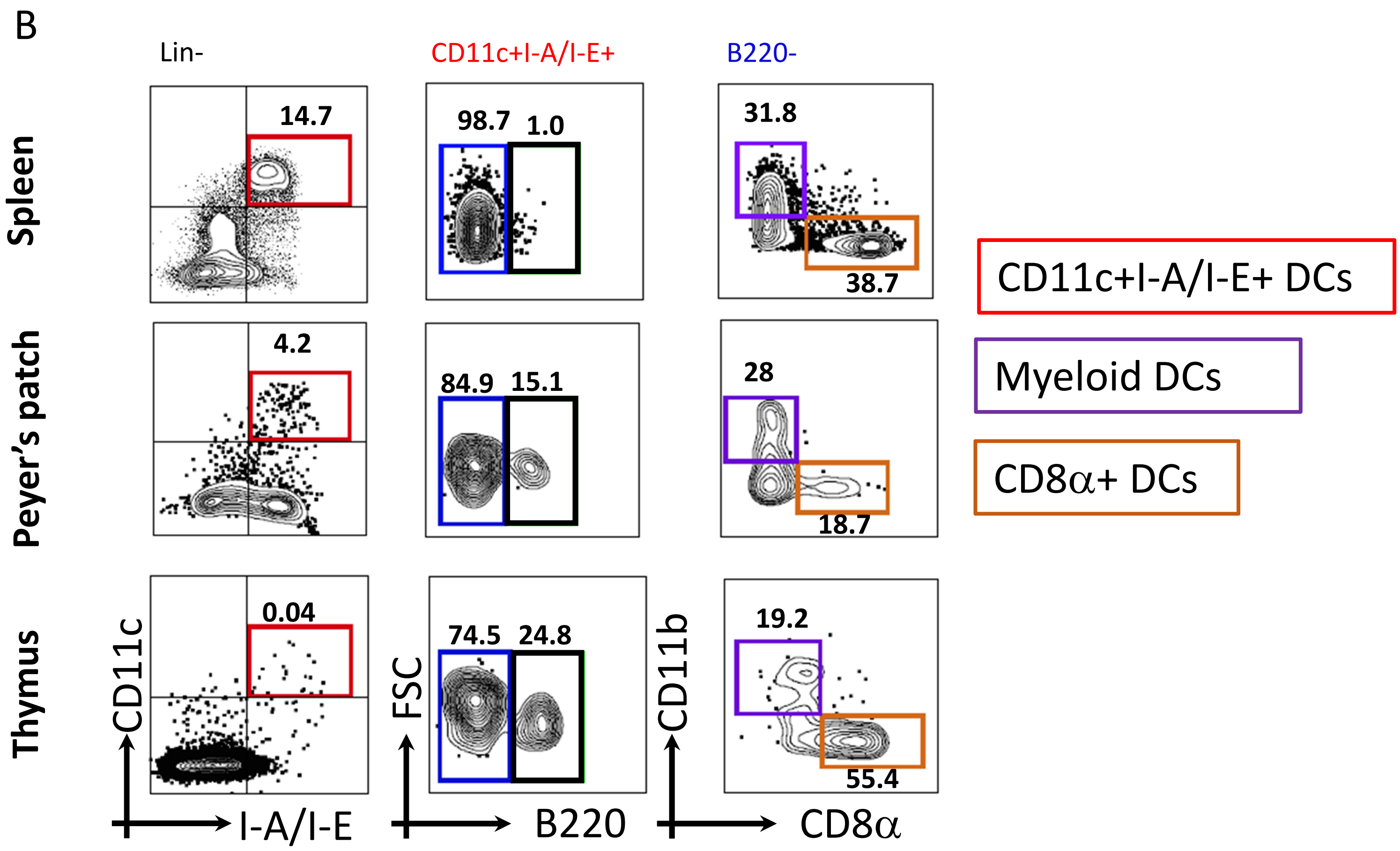

Supplementary FIGURE 1

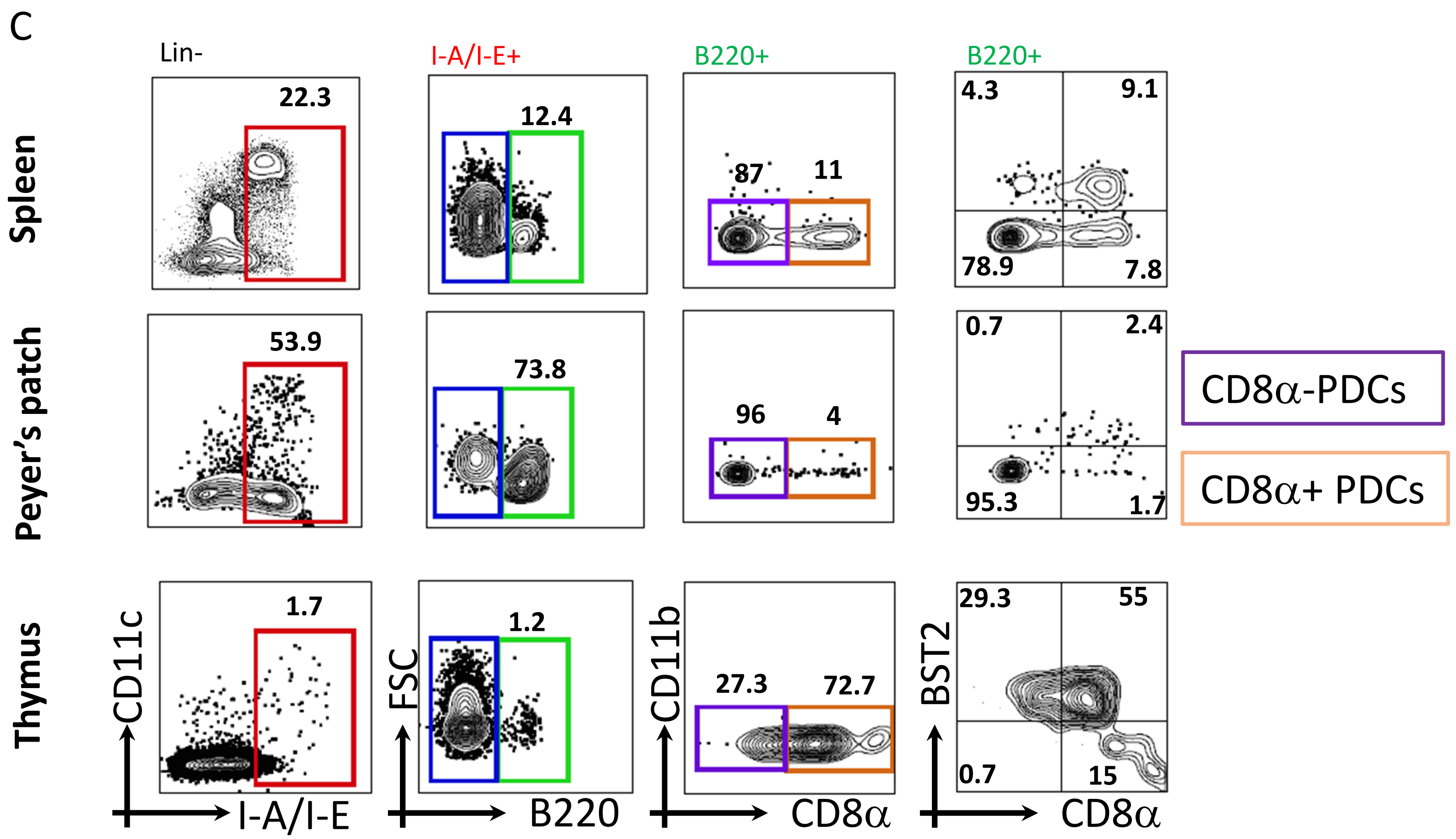

Supplementary FUGURE 1 | Flow cytometry gating strategy of dendritic cells in spleen, Peyer's patches, and thymus. **(A)** Gating strategy of lin1-(CD3^{neg}NK1.1^{neg}Ly6-G^{neg}CD19^{neg}) in spleen, Peyer's patches, and thymus. **(B)** Gating strategy of CD11c+I-A/I-E+DCs and subsets, myeloid DCs (B220^{neg}CD11b^{+}CD8α^{neg}), and CD8+DCs (B220^{neg}CD11b^{neg}CD8α^{+}) in spleen, Peyer's patches, and thymus. (**c**) Gating strategy of CD8α+/- PDCs (I-A/I-E^{+}B220^{+}) in spleen, Peyer's patches, and thymus.

Supplementary FIGURE. 2

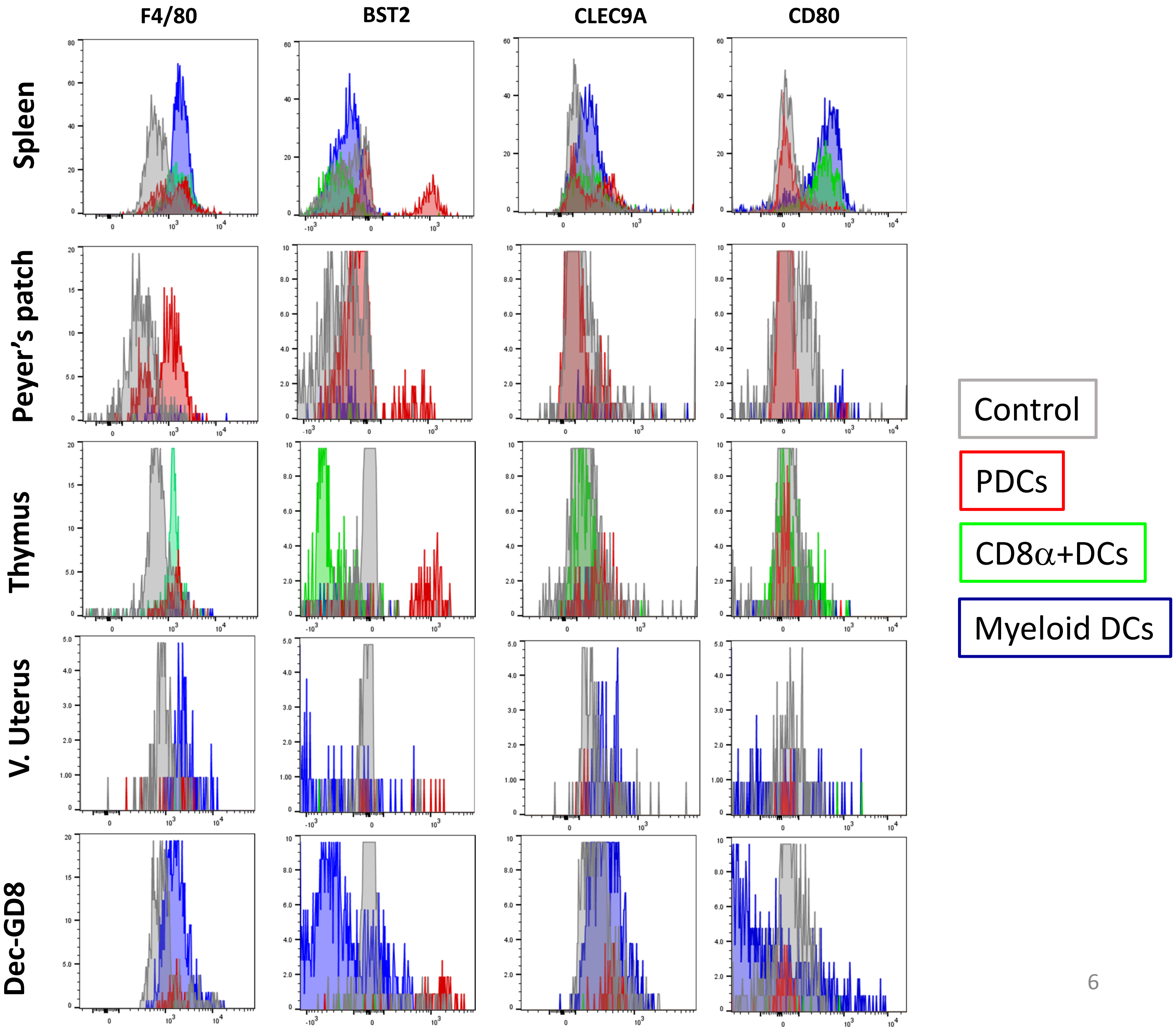

Supplementary FIGURE. 2

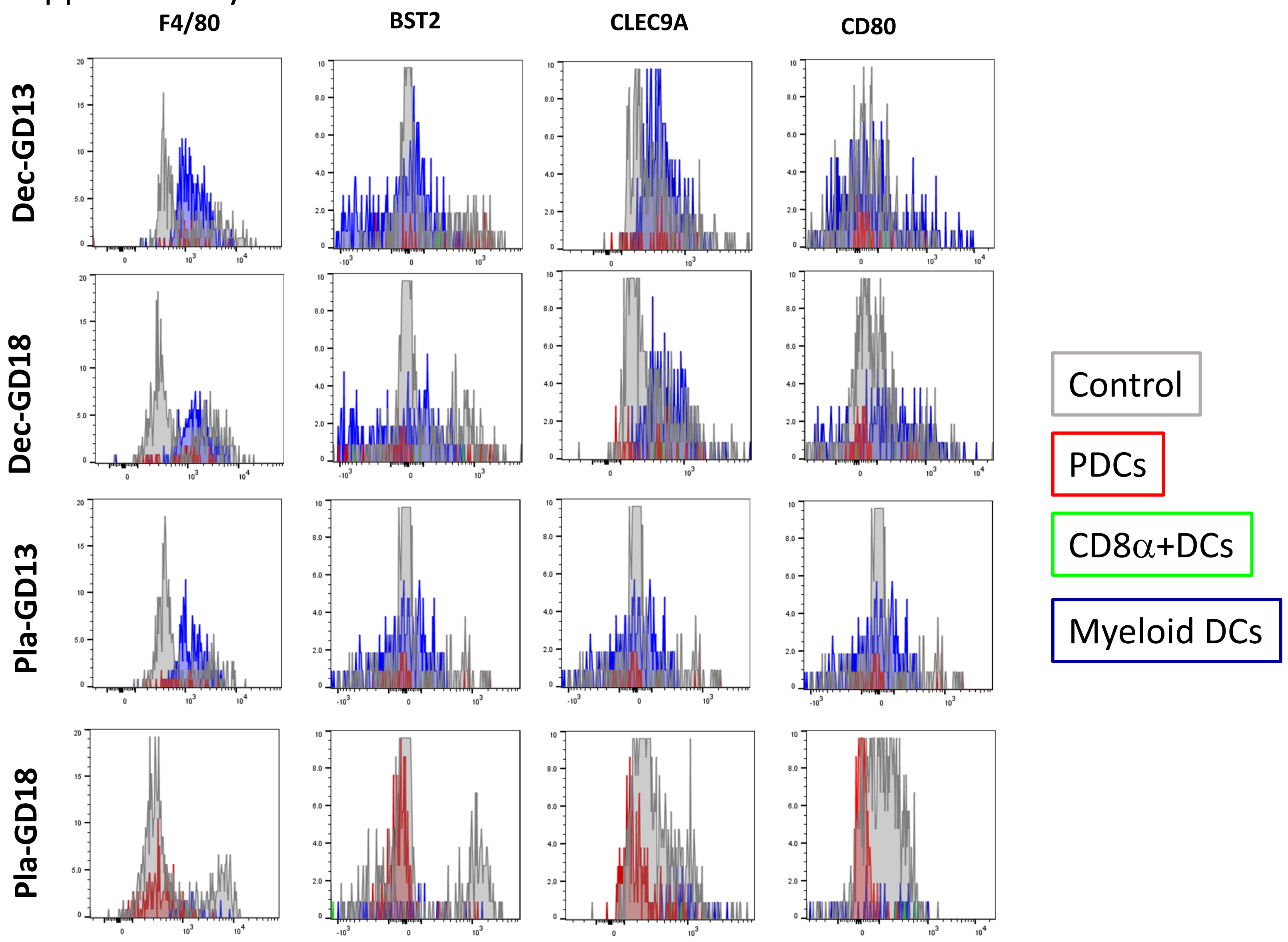

Supplementary FIGURE 2 | Comparison of surface makers (F4/80, BST2, CLEC9A, and CD80) expression among PDCs, CD8+ DCs, and myeloid DCs population in murine spleen, Peyer's patches, thymus, virgin uterus, decidua and placenta across pregnancy. NK1.1-/CD3- population used as control.

Supplementary FIGURE. 3

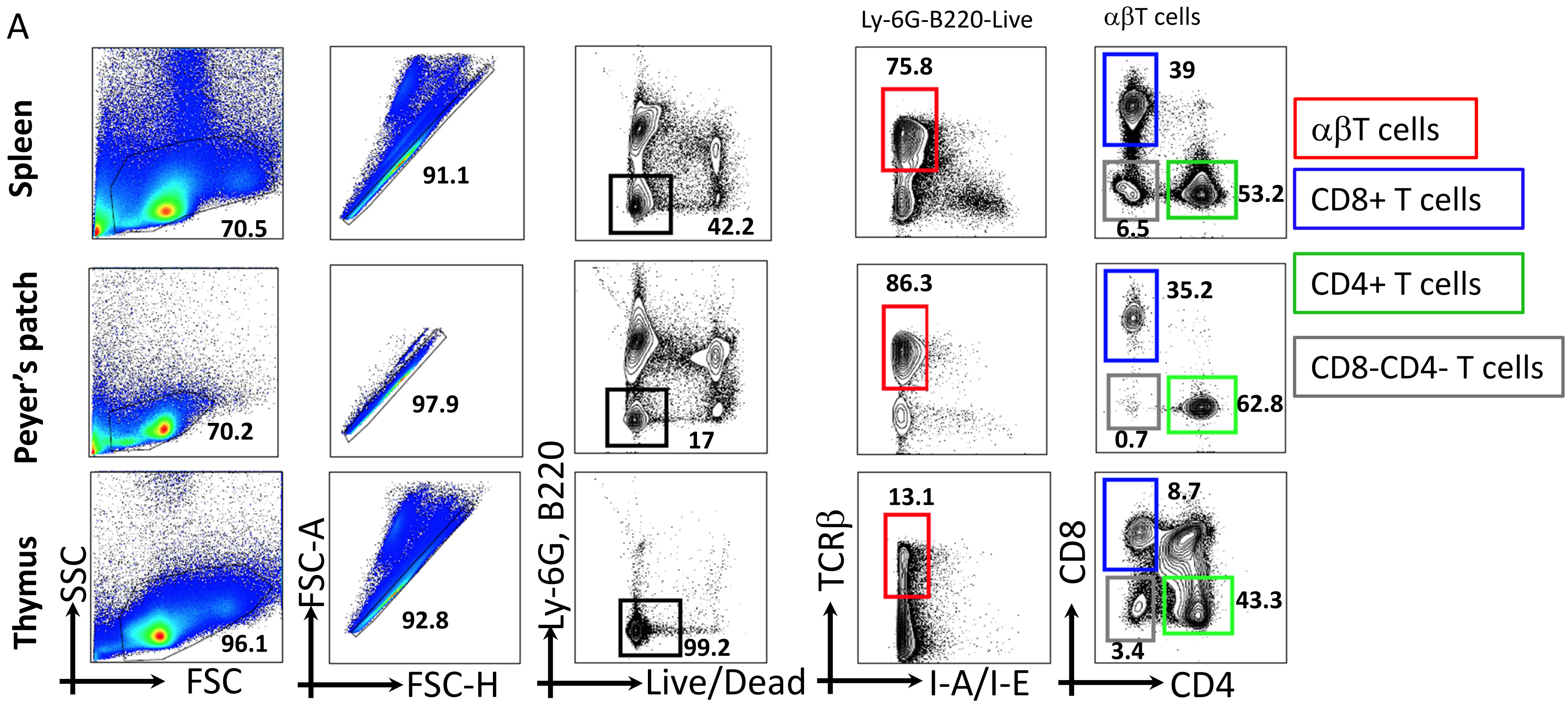

Supplementary FIGURE. 3

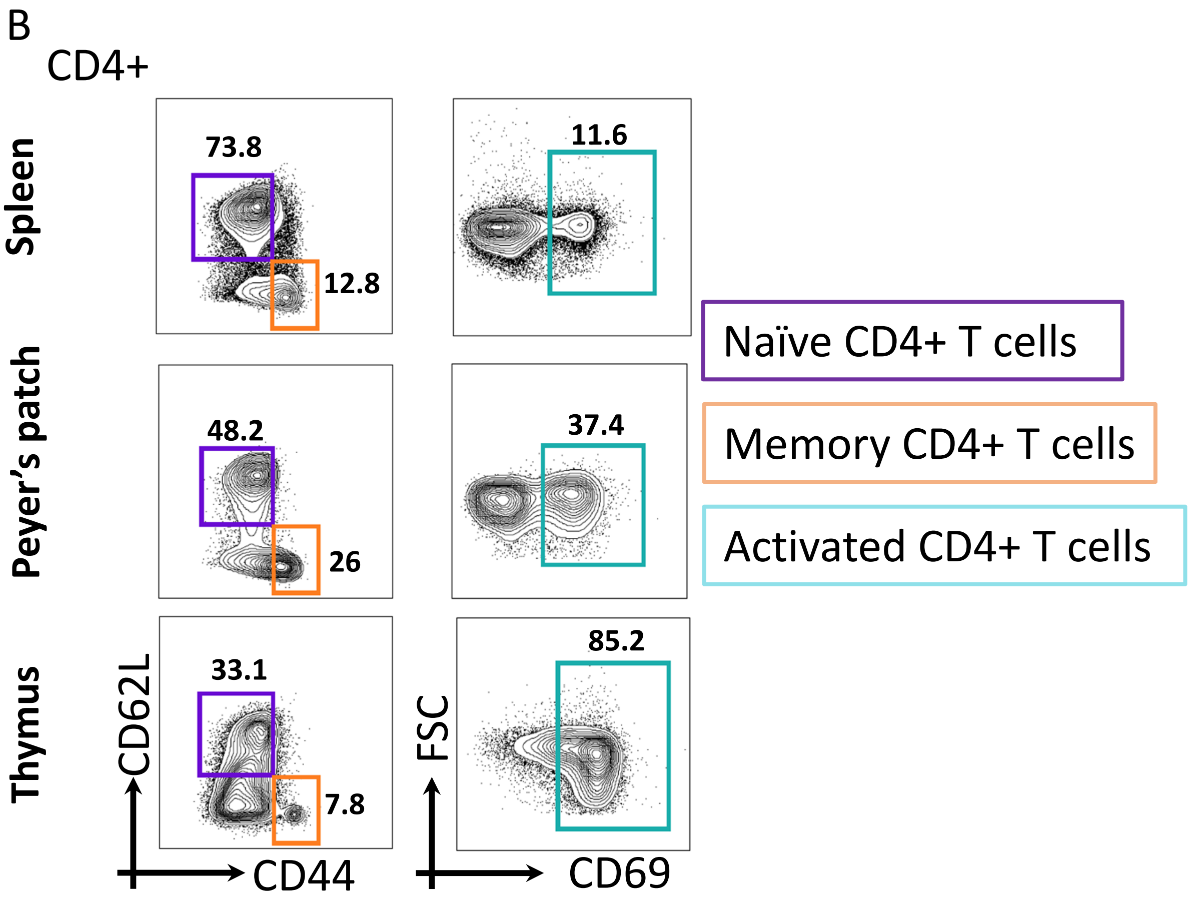

Supplementary FIGURE. 3

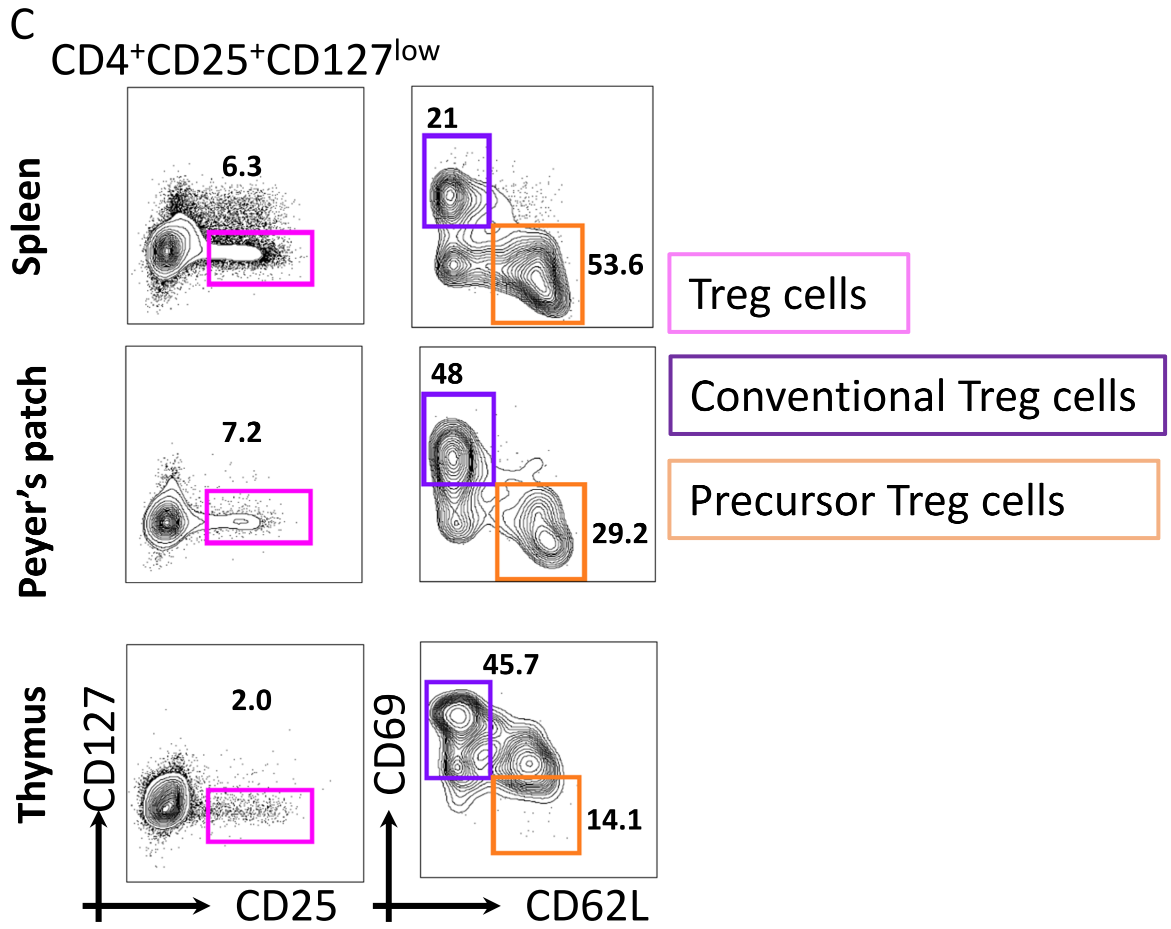

Supplementary FIGURE. 3

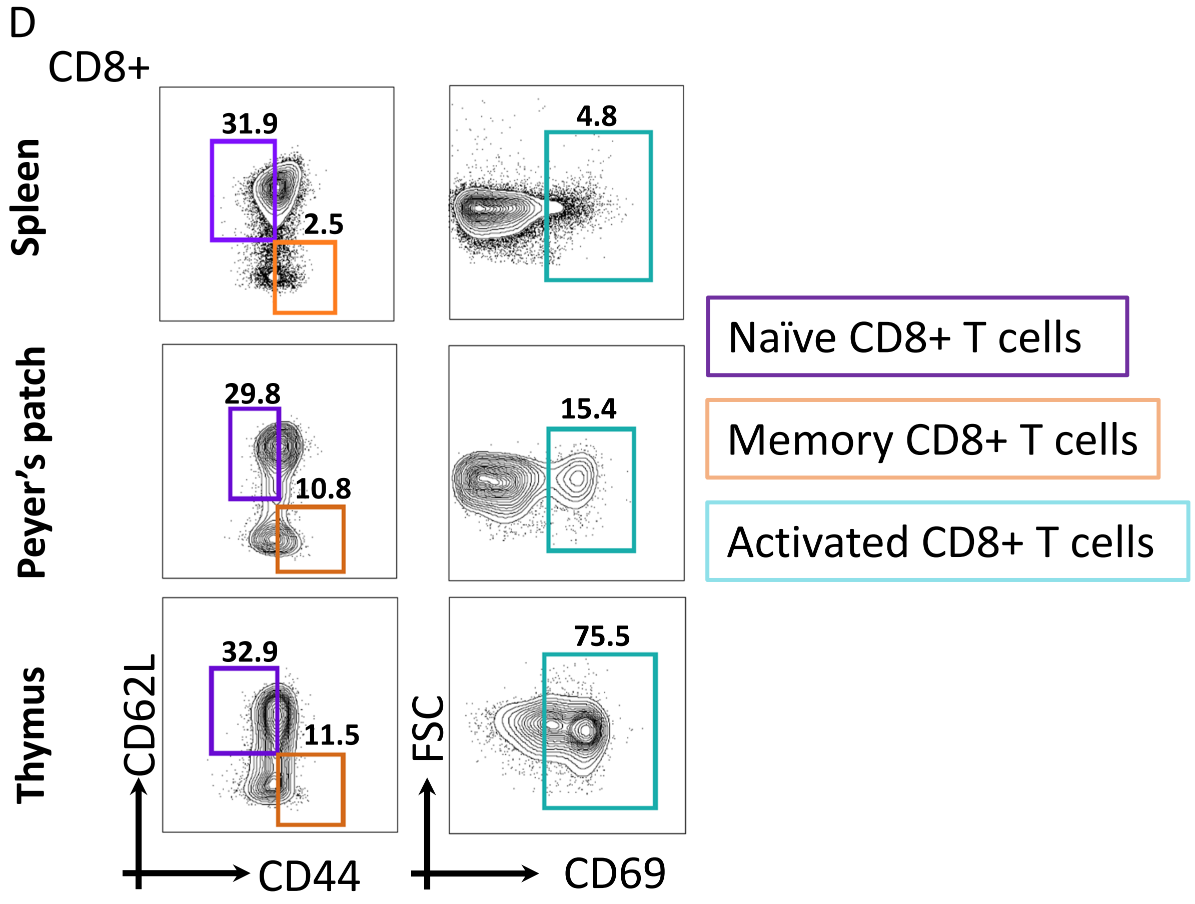

Supplementary FIGURE 3 | Flow cytometry gating strategy of T cells in spleen, Peyer's patches, and thymus. **(A)** Gating strategy of CD4+, CD8+ and CD8-CD4- T cells (pregated on lin2-: Ly6G^{neg}B220^{neg}I-A/I-E^{neg}, TCRβ^{+}). **(B)** Gating strategy of subsets of CD4+ T cell subsets. (**C**) Gating strategy of Treg cells and its subsets, conventional and precursor Treg cells. **(D)** Gating strategy of CD8+ T cell subsets.

Supplementary FIGURE 4

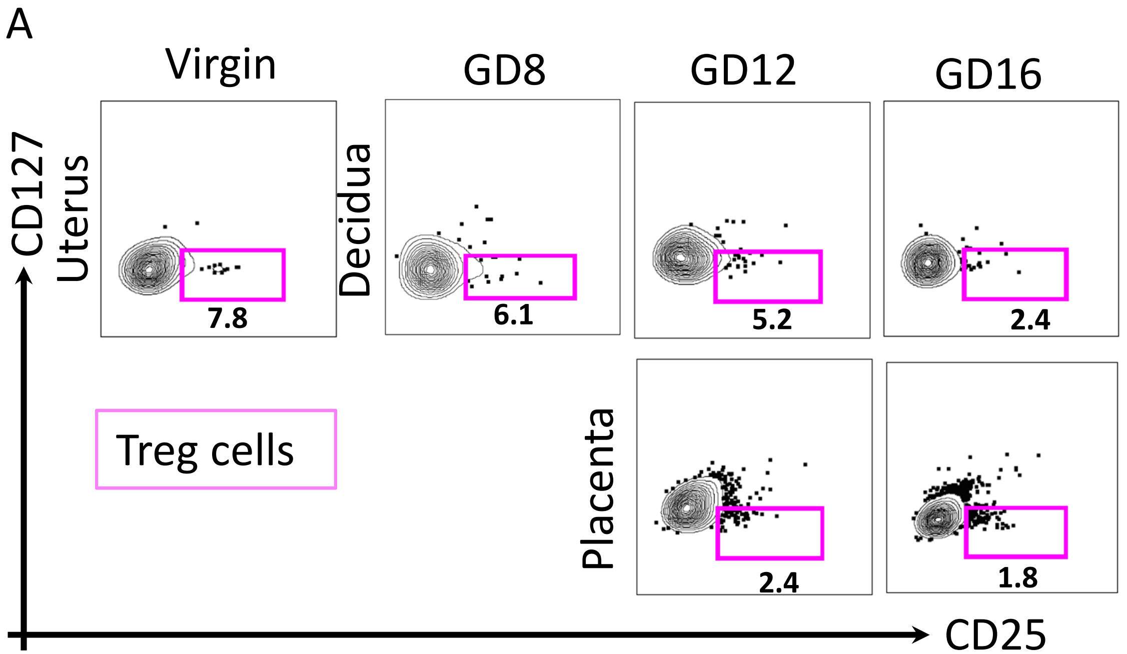

Supplementary FIGURE 4

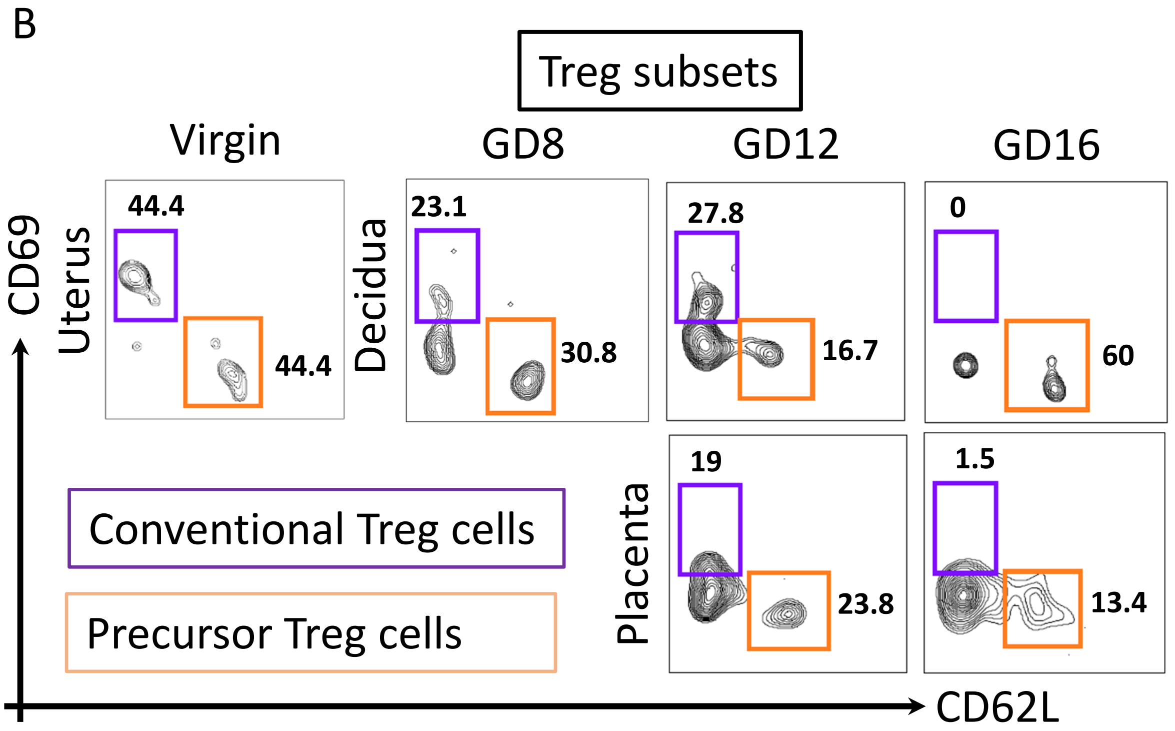

Supplementary FIGURE 4 | Gating strategy of Treg cells and subsets in murine virgin uterus, decidua and placenta across pregnancy. **(A and B)** Treg cells were defined as lin2-TCRβ^{+}CD4^{+}CD25^{+}CD127^{low}, conventional Treg cells and precursor Treg cells were defined as CD69^{+}CD62L^{neg} and CD69^{neg}CD62L^{+}, respectively.

Supplementary FIGURE 5

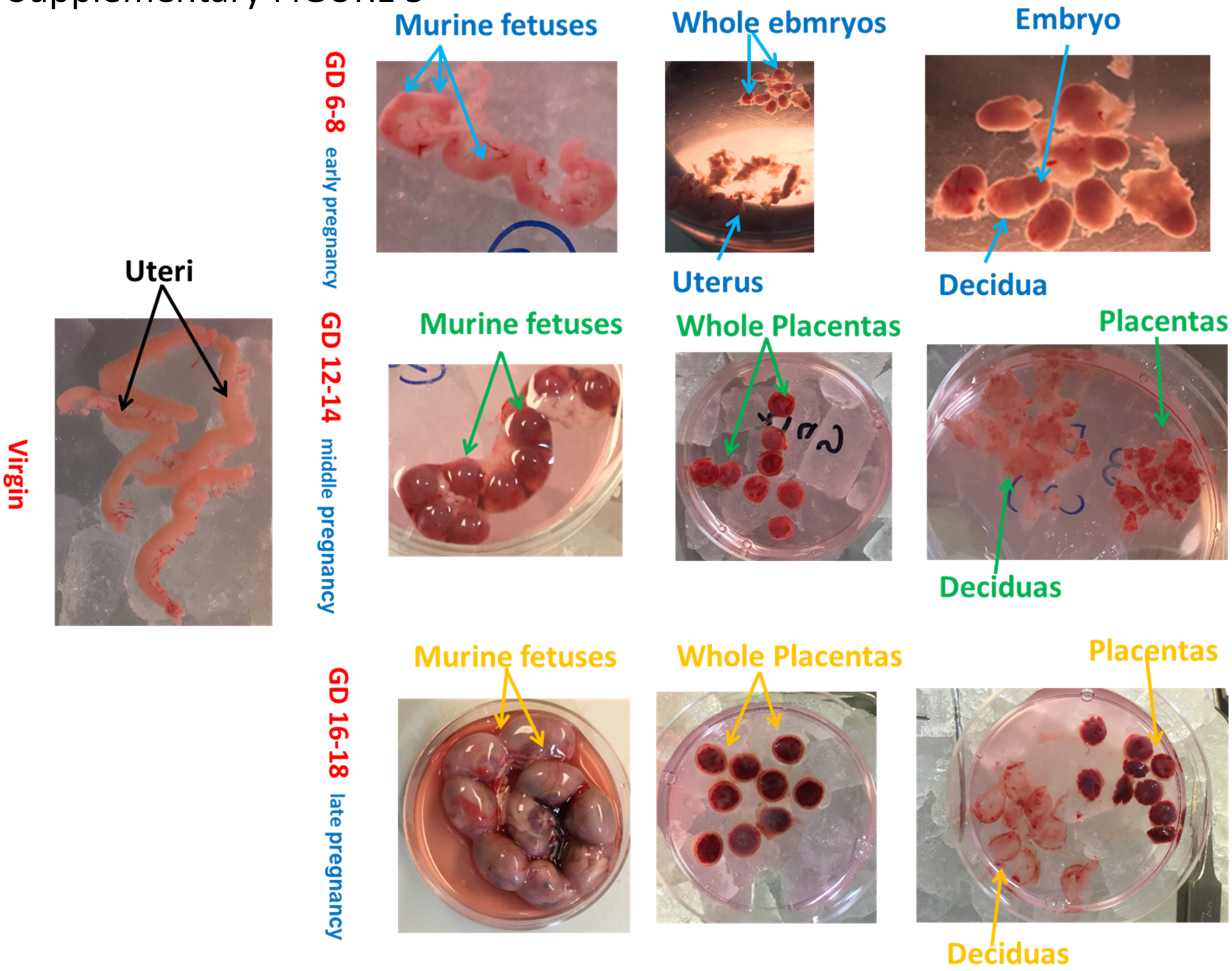

Supplementary FIGURE 5 | Representative pictures of tissue dissection for murine uteri (virgin), embryos (GD6-8), decidua and placenta at GD6-8 (early pregnancy), 12-14 (middle pregnancy), 16-18 (late pregnancy).

Supplementary Table 1: Groups of I-A/I-E^{+} clusters (Figure 6B)

Group	Clusters	Characterization
A	1, 2, 4, 5	High to median levels of CLEC9A, CD209, CD80, lower to median levels of BST2, B220, CD14, CD8a, and varied CD11b, F4/80, CD11c
B	7, 8, 11, 14	High expression of BST2, B220, CD14, median expression of CD209 and CD80, low expression of CD8a, CLEC9A, varied expression of CD11b, F4/80, CD11c
C	9	High expression of BST2, B220, CD14, median expression of CD80, low expression of CD8a, CLEC9A, CD209, CD11b, F4/80, CD11c
D	10, 12, 13, 15	High expression of BST2, B220, CD14, CLEC9A, CD209, median expression of CD80, low expression of CD11b, varied expression of F4/80 and CD11c
E	3, 6	High expression of CD11c, F4/80, CD11b, CLEC9A, CD209, low expression of CD14, CD8a, CD80, B220, and varied expression of BST2
F	16	High expression of CD8a, CD14, CD209, median expressions of BST2, B220, CD80, low expressions of CD11c, F4/80, CD11b, CLEC9A.

Supplementary Table 2: Groups of TCRβ^{+} clusters (Figure 7B)

Group	Clusters	Characterization
A	1, 2,	High expressions of CD44, CD8a, CD62L, low expressions of CD4, CD127, CD25, CD196, CD69, and high or low expression of CD122.
B	3, 5, 8	High to median expressions of CD196, CD122, low expressions of CD4, CD62L, CD127, CD25, and varied expressions of CD8a and CD44, CD196.
C	9, 13	High expression of CD4, CD44, median expression of CD122, and low expression of CD8a, CD62L, CD127, CD25, CD196, CD69.
D	4, 6	High expressions of CD4 and CD62L, median expression of CD122, low expression of CD44, CD8a, CD127, CD25, CD69, and high or low expression of CD196.
E	7, 10, 11, 12	High to median expressions of CD127, CD25, CD196, CD69, CD122, low expressions of CD4, CD8a, CD62L, and high or low expression of CD44.

Supplementary Table 3: Number of animals in each figure.

	# of Animals						
**Figures\Tissues**	**Virgin**	**Decidua GD6-8**	**Decidua GD12-14**	**Decidua GD16-18**	**Placenta GD12-14**	**Placenta GD16-18**	**Embryo**
Fig. 1D (CD11c+I-A/I-E+DCs)	16	14	15	15	14	15	14
Fig. 1E (Myeloid DCs)	16	14	15	15	15	15	14
Fig. 1F (CD8αDCs)	15	14	15	15	15	15	14
Fig. 2C (Plasmacytoid DCs)	15	14	13	15	13	15	14
Fig. 2D (CD8α-PDCs)	16	14	14	16	14	16	14
Fig. 2E (CD8α+PDCs)	16	14	14	16	14	16	13
Fig. 3D (αβT cells)	15	14	14	11	14	11	14
Fig. 3E (CD8+T cells)	15	14	14	12	13	12	14
Fig. 3F (CD4+ T cells)	15	14	14	12	14	12	14
Fig. 3G (CD4-CD8- T cells)	15	14	14	12	14	12	14
Fig. 4C (Naive CD4+ T cells)	15	14	14	12	13	12	14
Fig. 4D (Memory CD4+ T cells)	15	14	13	12	13	12	14
Fig. 4E (Activated CD4+ T cells)	15	14	14	12	14	12	14
Fig. 4F (Treg cells)	15	14	13	12	13	12	14
Fig. 4G (Conv Treg cells)	14	14	11	12	10	11	14
Fig. 4H (Precursor Treg cells)	14	13	11	13	11	13	13
Fig. 5C (Naive CD8+ T cells)	15	14	14	12	14	12	13
Fig. 5D (Memory CD8+ T cells)	15	14	14	12	14	12	13
Fig. 5E (Activated CD8+ T cells)	15	14	14	12	14	11	14

Supplementary Table 4. Related to Figure 1. Statistical comparisons for CD11c+I-A/I-E+DCs and subsets.

**Population**	**ANOVA**			**Tukey's post-hoc**			
	**F value**	**D.F**	**p-value**	**Comparisons**	**p-value**	**Significant**	**Summary**
CD11c+I-A/I-E+DCs (Fig. 1D)	15.3	3,56	**<0.0001**	V. Uterus vs. Decidua GD6-8	**<0.0001**	**Yes**	****
				V. Uterus vs. Decidua GD12-14	0.2634	No	ns
				V. Uterus vs. Decidua GD16-18	0.9941	No	ns
				Decidua GD6-8 vs. Decidua GD12-14	**0.0005**	**Yes**	***
				Decidua GD6-8 vs. Decidua GD16-18	**<0.0001**	**Yes**	****
				Decidua GD12-14 vs. Decidua GD16-18	0.4065	No	ns
	16.9	3,55	**<0.0001**	V. Uterus vs. Placenta GD12-14	0.8632	No	ns
				V. Uterus vs. Placenta GD16-18	0.8866	No	ns
				V. Uterus vs. Embryo GD6-8	**<0.0001**	**Yes**	****
				Placenta GD12-14 vs. Placenta GD16-18	0.9999	No	ns
				Placenta GD12-14 vs. Embryo GD6-8	**<0.0001**	**Yes**	****
				Placenta GD16-18 vs. Embryo GD6-8	**<0.0001**	**Yes**	****
Myeloid DCs (Fig. 1E)	7.5	3,56	**0.0003**	V. Uterus vs. Decidua GD6-8	**0.03**	**Yes**	*
				V. Uterus vs. Decidua GD12-14	0.9951	No	ns
				V. Uterus vs. Decidua GD16-18	**0.0039**	**Yes**	**
				Decidua GD6-8 vs. Decidua GD12-14	0.0183	**Yes**	*
				Decidua GD6-8 vs. Decidua GD16-18	0.9128	No	ns
				Decidua GD12-14 vs. Decidua GD16-18	**0.0022**	**Yes**	**
	5.7	3,56	0.0018	V. Uterus vs. Placenta GD12-14	0.4478	No	ns
				V. Uterus vs. Placenta GD16-18	0.1488	No	ns
				V. Uterus vs. Embryo GD6-8	**0.0008**	**Yes**	***
				Placenta GD12-14 vs. Placenta GD16-18	0.9151	No	ns
				Placenta GD12-14 vs. Embryo GD6-8	0.061	No	ns
				Placenta GD16-18 vs. Embryo GD6-8	0.2292	No	ns
CD8αDCs (Fig. 1F)	1.5	3,55	0.2234	V. Uterus vs. Decidua GD6-8	0.404	No	ns
				V. Uterus vs. Decidua GD12-14	0.9842	No	ns
				V. Uterus vs. Decidua GD16-18	0.9995	No	ns
				Decidua GD6-8 vs. Decidua GD12-14	0.2314	No	ns
				Decidua GD6-8 vs. Decidua GD16-18	0.3432	No	ns
				Decidua GD12-14 vs. Decidua GD16-18	0.9949	No	ns
	1.1	3,55	0.3743	V. Uterus vs. Placenta GD12-14	0.9296	No	ns
				V. Uterus vs. Placenta GD16-18	0.536	No	ns
				V. Uterus vs. Embryo GD6-8	0.989	No	ns
				Placenta GD12-14 vs. Placenta GD16-18	0.8793	No	ns
				Placenta GD12-14 vs. Embryo GD6-8	0.7989	No	ns
				Placenta GD16-18 vs. Embryo GD6-8	0.3645	No	ns

Supplementary Table 5. Related to Figure 2. Statistical comparisons for Plasmacytoid DCs and subsets.

Population	ANOVA			Tukey's post-hoc			
	F value	D.F	p-value	Comparisons	p-value	Significant	Summary
Plasmacytoid DCs (Fig. 2C)	10.03	3,53	**<0.0001**	V. Uterus vs. Decidua GD6-8	**0.0012**	**Yes**	**
				V. Uterus vs. Decidua GD12-14	**0.0108**	**Yes**	*
				V. Uterus vs. Decidua GD16-18	**<0.0001**	**Yes**	****
				Decidua GD6-8 vs. Decidua GD12-14	0.9123	No	ns
				Decidua GD6-8 vs. Decidua GD16-18	0.6595	No	ns
				Decidua GD12-14 vs. Decidua GD16-18	0.2857	No	ns
	3.557	3,53	**0.0202**	V. Uterus vs. Placenta GD12-14	0.1214	No	ns
				V. Uterus vs. Placenta GD16-18	0.1157	No	ns
				V. Uterus vs. Embryo GD6-8	**0.017**	**Yes**	*
				Placenta GD12-14 vs. Placenta GD16-18	>0.9999	No	ns
				Placenta GD12-14 vs. Embryo GD6-8	0.8776	No	ns
				Placenta GD16-18 vs. Embryo GD6-8	0.8377	No	ns
CD8α-PDCs (Fig. 2D)	6.764	3,56	**0.0006**	V. Uterus vs. Decidua GD6-8	0.2949	No	ns
				V. Uterus vs. Decidua GD12-14	0.2765	No	ns
				V. Uterus vs. Decidua GD16-18	0.0992	No	ns
				Decidua GD6-8 vs. Decidua GD12-14	**0.0053**	**Yes**	**
				Decidua GD6-8 vs. Decidua GD16-18	0.9605	No	ns
				Decidua GD12-14 vs. Decidua GD16-18	**0.0008**	**Yes**	***
	8.201	3,56	0.0001	V. Uterus vs. Placenta GD12-14	**0.0165**	**Yes**	*
				V. Uterus vs. Placenta GD16-18	0.9152	No	ns
				V. Uterus vs. Embryo GD6-8	0.2047	No	ns
				Placenta GD12-14 vs. Placenta GD16-18	0.0791	No	ns
				Placenta GD12-14 vs. Embryo GD6-8	**<0.0001**	**Yes**	****
				Placenta GD16-18 vs. Embryo GD6-8	0.054	No	ns
CD8α+PDCs (Fig. 2E)	3.593	3,56	0.019	V. Uterus vs. Decidua GD6-8	0.8586	No	ns
				V. Uterus vs. Decidua GD12-14	0.087	No	ns
				V. Uterus vs. Decidua GD16-18	0.9997	No	ns
				Decidua GD6-8 vs. Decidua GD12-14	**0.0157**	**Yes**	*
				Decidua GD6-8 vs. Decidua GD16-18	0.8166	No	ns
				Decidua GD12-14 vs. Decidua GD16-18	0.1052	No	ns
	9.748	3,55	**<0.0001**	V. Uterus vs. Placenta GD12-14	**0.0003**	**Yes**	***
				V. Uterus vs. Placenta GD16-18	**0.0302**	**Yes**	*
				V. Uterus vs. Embryo GD6-8	0.9817	No	ns
				Placenta GD12-14 vs. Placenta GD16-18	0.3938	No	ns
				Placenta GD12-14 vs. Embryo GD6-8	**0.0002**	**Yes**	***
				Placenta GD16-18 vs. Embryo GD6-8	**0.0167**	**Yes**	*

Supplementary Table 6. Related to Figure 3. Statistical comparisons for T cells and subsets.

Population	ANOVA			Tukey's post-hoc			
	F value	D.F	p-value	Comparisons	p-value	Significant	Summary
αβT cells (Fig. 3D)	18.06	3,50	**<0.0001**	V. Uterus vs. Decidua GD6-8	0.4731	No	ns
				V. Uterus vs. Decidua GD12-14	**0.0473**	**Yes**	*
				V. Uterus vs. Decidua GD16-18	**<0.0001**	**Yes**	****
				Decidua GD6-8 vs. Decidua GD12-14	**0.001**	**Yes**	***
				Decidua GD6-8 vs. Decidua GD16-18	**<0.0001**	**Yes**	****
				Decidua GD12-14 vs. Decidua GD16-18	**0.0207**	**Yes**	*
	2.691	3,50	0.0561	V. Uterus vs. Placenta GD12-14	0.4742	No	ns
				V. Uterus vs. Placenta GD16-18	0.8282	No	ns
				V. Uterus vs. Embryo GD6-8	0.6205	No	ns
				Placenta GD12-14 vs. Placenta GD16-18	0.1429	No	ns
				Placenta GD12-14 vs. Embryo GD6-8	0.0545	No	ns
				Placenta GD16-18 vs. Embryo GD6-8	0.9923	No	ns
CD8+ T cells (Fig. 3E)	1.049	3,51	0.3788	V. Uterus vs. Decidua GD6-8	0.4624	No	ns
				V. Uterus vs. Decidua GD12-14	0.9999	No	ns
				V. Uterus vs. Decidua GD16-18	0.8502	No	ns
				Decidua GD6-8 vs. Decidua GD12-14	0.4367	No	ns
				Decidua GD6-8 vs. Decidua GD16-18	0.9337	No	ns
				Decidua GD12-14 vs. Decidua GD16-18	0.8245	No	ns
	2.507	3,50	0.0695	V. Uterus vs. Placenta GD12-14	0.9431	No	ns
				V. Uterus vs. Placenta GD16-18	**0.0563**	No	ns
				V. Uterus vs. Embryo GD6-8	0.5019	No	ns
				Placenta GD12-14 vs. Placenta GD16-18	0.2049	No	ns
				Placenta GD12-14 vs. Embryo GD6-8	0.8521	No	ns
				Placenta GD16-18 vs. Embryo GD6-8	0.6053	No	ns
CD4+ T cells (Fig. 3F)	6.688	3,51	0.0007	V. Uterus vs. Decidua GD6-8	**0.0006**	**Yes**	***
				V. Uterus vs. Decidua GD12-14	**0.0218**	**Yes**	*
				V. Uterus vs. Decidua GD16-18	**0.0136**	**Yes**	*
				Decidua GD6-8 vs. Decidua GD12-14	0.6295	No	ns
				Decidua GD6-8 vs. Decidua GD16-18	0.8246	No	ns
				Decidua GD12-14 vs. Decidua GD16-18	0.9912	No	ns
	4.753	3,51	0.0053	V. Uterus vs. Placenta GD12-14	**0.006**	**Yes**	**
				V. Uterus vs. Placenta GD16-18	0.4409	No	ns
				V. Uterus vs. Embryo GD6-8	**0.0264**	**Yes**	*
				Placenta GD12-14 vs. Placenta GD16-18	0.2966	No	ns
				Placenta GD12-14 vs. Embryo GD6-8	0.9516	No	ns
				Placenta GD16-18 vs. Embryo GD6-8	0.5875	No	ns
CD8-CD4- T cells (Fig. 3G)	3.722	3,51	0.017	V. Uterus vs. Decidua GD6-8	0.0665	No	ns
				V. Uterus vs. Decidua GD12-14	**0.0306**	**Yes**	*
				V. Uterus vs. Decidua GD16-18	0.0527	No	ns
				Decidua GD6-8 vs. Decidua GD12-14	0.989	No	ns
				Decidua GD6-8 vs. Decidua GD16-18	0.9972	No	ns
				Decidua GD12-14 vs. Decidua GD16-18	0.9996	No	ns
	6.148	3,51	0.0012	V. Uterus vs. Placenta GD12-14	**0.0008**	**Yes**	***
				V. Uterus vs. Placenta GD16-18	0.4187	No	ns
				V. Uterus vs. Embryo GD6-8	0.7658	No	ns
				Placenta GD12-14 vs. Placenta GD16-18	0.0934	No	ns
				Placenta GD12-14 vs. Embryo GD6-8	**0.0158**	**Yes**	*
				Placenta GD16-18 vs. Embryo GD6-8	**0.9307**	**No**	**ns**

Supplementary Table 7. Related to Figure 4. Statistical comparisons for CD4+T cells and subsets.

Population	ANOVA			Tukey's post-hoc			
	F value	D.F	p-value	Comparisons	p-value	Significant	Summary
Naïve CD4+ T cells (Fig. 4C)	3.212	3,51	**0.0305**	V. Uterus vs. Decidua GD6-8	0.6715	No	ns
				V. Uterus vs. Decidua GD12-14	0.8036	No	ns
				V. Uterus vs. Decidua GD16-18	0.2303	No	ns
				Decidua GD6-8 vs. Decidua GD12-14	0.2013	No	ns
				Decidua GD6-8 vs. Decidua GD16-18	**0.023**	**Yes**	*
				Decidua GD12-14 vs. Decidua GD16-18	0.7249	No	ns
	11.79	3,50	**<0.0001**	V. Uterus vs. Placenta GD12-14	**<0.0001**	**Yes**	****
				V. Uterus vs. Placenta GD16-18	**0.0002**	**Yes**	***
				V. Uterus vs. Embryo GD6-8	0.5483	No	ns
				Placenta GD12-14 vs. Placenta GD16-18	0.9979	No	ns
				Placenta GD12-14 vs. Embryo GD6-8	**0.0048**	**Yes**	**
				Placenta GD16-18 vs. Embryo GD6-8	**0.0101**	**Yes**	*
Memory CD4+ T cells (Fig. 4D)	1.025	3,50	0.3895	V. Uterus vs. Decidua GD6-8	0.9473	No	ns
				V. Uterus vs. Decidua GD12-14	0.4986	No	ns
				V. Uterus vs. Decidua GD16-18	0.993	No	ns
				Decidua GD6-8 vs. Decidua GD12-14	0.8244	No	ns
				Decidua GD6-8 vs. Decidua GD16-18	0.8625	No	ns
				Decidua GD12-14 vs. Decidua GD16-18	0.3895	No	ns
	4.106	3,50	**0.0111**	V. Uterus vs. Placenta GD12-14	0.329	No	ns
				V. Uterus vs. Placenta GD16-18	**0.0067**	**Yes**	**
				V. Uterus vs. Embryo GD6-8	0.1137	No	ns
				Placenta GD12-14 vs. Placenta GD16-18	0.342	No	ns
				Placenta GD12-14 vs. Embryo GD6-8	0.9519	No	ns
				Placenta GD16-18 vs. Embryo GD6-8	0.6307	No	ns
Activated CD4+ T cells (Fig. 4E)	7.537	3,51	**0.0003**	V. Uterus vs. Decidua GD6-8	0.2566	No	ns
				V. Uterus vs. Decidua GD12-14	**0.0348**	**Yes**	*
				V. Uterus vs. Decidua GD16-18	**0.0001**	**Yes**	***
				Decidua GD6-8 vs. Decidua GD12-14	0.7916	No	ns
				Decidua GD6-8 vs. Decidua GD16-18	**0.033**	**Yes**	*
				Decidua GD12-14 vs. Decidua GD16-18	0.2258	No	ns
	37.71	3,51	**<0.0001**	V. Uterus vs. Placenta GD12-14	**<0.0001**	**Yes**	****
				V. Uterus vs. Placenta GD16-18	**<0.0001**	**Yes**	****
				V. Uterus vs. Embryo GD6-8	**0.0002**	**Yes**	***
				Placenta GD12-14 vs. Placenta GD16-18	0.3431	No	ns
				Placenta GD12-14 vs. Embryo GD6-8	**0.0035**	**Yes**	**
				Placenta GD16-18 vs. Embryo GD6-8	**<0.0001**	**Yes**	****
Treg cells (Fig. 4F)	4.5	3,50	**0.0072**	V. Uterus vs. Decidua GD6-8	0.0784	No	ns
				V. Uterus vs. Decidua GD12-14	0.1203	No	ns
				V. Uterus vs. Decidua GD16-18	**0.005**	**Yes**	**
				Decidua GD6-8 vs. Decidua GD12-14	0.9988	No	ns
				Decidua GD6-8 vs. Decidua GD16-18	0.666	No	ns
				Decidua GD12-14 vs. Decidua GD16-18	0.5895	No	ns
	9.191	3,50	**<0.0001**	V. Uterus vs. Placenta GD12-14	**0.0001**	**Yes**	***
				V. Uterus vs. Placenta GD16-18	**0.0005**	**Yes**	***
				V. Uterus vs. Embryo GD6-8	**0.0264**	**Yes**	*
				Placenta GD12-14 vs. Placenta GD16-18	0.9948	No	ns
				Placenta GD12-14 vs. Embryo GD6-8	0.3059	No	ns
				Placenta GD16-18 vs. Embryo GD6-8	0.4624	No	ns
Conventional Treg cells (Fig. 4G)	3.572	3,47	**0.0208**	V. Uterus vs. Decidua GD6-8	0.9826	No	ns
				V. Uterus vs. Decidua GD12-14	0.8381	No	ns
				V. Uterus vs. Decidua GD16-18	**0.0203**	**Yes**	*
				Decidua GD6-8 vs. Decidua GD12-14	0.9615	No	ns
				Decidua GD6-8 vs. Decidua GD16-18	**0.0493**	**Yes**	*
				Decidua GD12-14 vs. Decidua GD16-18	0.1863	No	ns
	19.25	3,45	**<0.0001**	V. Uterus vs. Placenta GD12-14	**<0.0001**	**Yes**	****
				V. Uterus vs. Placenta GD16-18	**<0.0001**	**Yes**	****
				V. Uterus vs. Embryo GD6-8	**0.0378**	**Yes**	*
				Placenta GD12-14 vs. Placenta GD16-18	0.9548	No	ns
				Placenta GD12-14 vs. Embryo GD6-8	**0.0084**	**Yes**	**
				Placenta GD16-18 vs. Embryo GD6-8	**0.0012**	**Yes**	**
Precursor Treg cells (Fig. 4H)	2.492	3,47	0.0716	V. Uterus vs. Decidua GD6-8	0.0941	No	ns
				V. Uterus vs. Decidua GD12-14	0.4751	No	ns
				V. Uterus vs. Decidua GD16-18	0.9976	No	ns
				Decidua GD6-8 vs. Decidua GD12-14	0.847	No	ns
				Decidua GD6-8 vs. Decidua GD16-18	0.1508	No	ns
				Decidua GD12-14 vs. Decidua GD16-18	0.5994	No	ns
	0.1348	3,47	0.9388	V. Uterus vs. Placenta GD12-14	0.972	No	ns
				V. Uterus vs. Placenta GD16-18	0.9943	No	ns
				V. Uterus vs. Embryo GD6-8	0.9984	No	ns
				Placenta GD12-14 vs. Placenta GD16-18	0.9975	No	ns
				Placenta GD12-14 vs. Embryo GD6-8	0.9364	No	ns
				Placenta GD16-18 vs. Embryo GD6-8	0.9765	No	ns

Supplementary Table 8. Related to Figure 5. Statistical comparisons for Plasmacytoid DCs and subsets.

Population	ANOVA			Tukey's post-hoc			
	**F value**	**D.F**	**p-value**	**Comparisons**	**p-value**	**Significant**	**Summary**
Naïve CD8+ T cells (Fig. 5C)	4.651	3,51	**0.006**	V. Uterus vs. Decidua GD6-8	0.9349	No	ns
				V. Uterus vs. Decidua GD12-14	0.99	No	ns
				V. Uterus vs. Decidua GD16-18	**0.0228**	**Yes**	*
				Decidua GD6-8 vs. Decidua GD12-14	0.8156	No	ns
				Decidua GD6-8 vs. Decidua GD16-18	**0.0056**	**Yes**	**
				Decidua GD12-14 vs. Decidua GD16-18	0.0526	No	ns
	2.352	3,50	0.0834	V. Uterus vs. Placenta GD12-14	0.4641	No	ns
				V. Uterus vs. Placenta GD16-18	0.155	No	ns
				V. Uterus vs. Embryo GD6-8	0.9995	No	ns
				Placenta GD12-14 vs. Placenta GD16-18	0.889	No	ns
				Placenta GD12-14 vs. Embryo GD6-8	0.4298	No	ns
				Placenta GD16-18 vs. Embryo GD6-8	0.1443	No	ns
Memory CD8+ T cell (Fig. 5D)	3.177	3,51	**0.0318**	V. Uterus vs. Decidua GD6-8	>0.9999	No	ns
				V. Uterus vs. Decidua GD12-14	0.8854	No	ns
				V. Uterus vs. Decidua GD16-18	**0.0436**	**Yes**	*
				Decidua GD6-8 vs. Decidua GD12-14	0.8707	No	ns
				Decidua GD6-8 vs. Decidua GD16-18	**0.0433**	**Yes**	*
				Decidua GD12-14 vs. Decidua GD16-18	0.2081	No	ns
	13.2	3,50	**<0.0001**	V. Uterus vs. Placenta GD12-14	**<0.0001**	**Yes**	****
				V. Uterus vs. Placenta GD16-18	**<0.0001**	**Yes**	****
				V. Uterus vs. Embryo GD6-8	**0.0107**	**Yes**	*
				Placenta GD12-14 vs. Placenta GD16-18	0.9952	No	ns
				Placenta GD12-14 vs. Embryo GD6-8	0.2104	No	ns
				Placenta GD16-18 vs. Embryo GD6-8	0.1558	No	ns
Activated CD8+ T cell(Fig. 5E)	5.465	3,50	**0.0025**	V. Uterus vs. Decidua GD6-8	0.7397	No	ns
				V. Uterus vs. Decidua GD12-14	0.7536	No	ns
				V. Uterus vs. Decidua GD16-18	**0.0017**	**Yes**	**
				Decidua GD6-8 vs. Decidua GD12-14	>0.9999	No	ns
				Decidua GD6-8 vs. Decidua GD16-18	**0.0279**	**Yes**	*
				Decidua GD12-14 vs. Decidua GD16-18	**0.0263**	**Yes**	*
	19.85	3,49	**<0.0001**	V. Uterus vs. Placenta GD12-14	**0.0001**	**Yes**	***
				V. Uterus vs. Placenta GD16-18	**<0.0001**	**Yes**	****
				V. Uterus vs. Embryo GD6-8	0.0916	No	ns
				Placenta GD12-14 vs. Placenta GD16-18	**0.02**	**Yes**	*
				Placenta GD12-14 vs. Embryo GD6-8	0.1243	No	ns
				Placenta GD16-18 vs. Embryo GD6-8	**<0.0001**	**Yes**	****
